# Dichotocejpins A–C: New Diketopiperazines from a Deep-Sea-Derived Fungus *Dichotomomyces cejpii* FS110

**DOI:** 10.3390/md14090164

**Published:** 2016-09-09

**Authors:** Zhen Fan, Zhang-Hua Sun, Zhong Liu, Yu-Chan Chen, Hong-Xin Liu, Hao-Hua Li, Wei-Min Zhang

**Affiliations:** 1 State Key Laboratory of Applied Microbiology Southern China, Guangdong Provincial Key Laboratory of Microbial Culture Collection and Application, Guangdong Open Laboratory of Applied Microbiology, Guangdong Institute of Microbiology, Guangzhou 510070, China; 15627860105@163.com (Z.F.); sunzh@gdim.cn (Z.-H.S.); yuchan2006@126.com (Y.-C.C.); hxinliu1225@163.com (H.-X.L.); hhli100@126.com (H.-H.L.); 2 Guangdong Provincial Key Laboratory of Bioengineering Medicine, National Engineering Research Center of Genetic Medicine, College of Life Science and Technology, Jinan University, Guangzhou 510632, China; tliuzh@jnu.edu.cn

**Keywords:** diketopiperazines, *Dichotomomyces cejpii*, deep-sea-derived fungus, cytotoxity, α-glucosidase

## Abstract

Three new diketopiperazines, dichotocejpins A–C (**1**–**3**), together with eight known analogues (**4**–**11**), were isolated from the culture of the deep-sea sediment derived fungus *Dichotomomyces cejpii* FS110. Their structures, including absolute configurations, were elucidated by a combination of HRESIMS, NMR, X-ray crystallography, and ECD calculations. Compounds **4**–**6**, **10**–**11** showed significant cytotoxic activities against MCF-7, NCI-H460, HepG-2, and SF-268 tumor cell lines. Compound **1** exhibited excellent inhibitory activity against *α*-glucosidase with an IC_50_ of 138 μM.

## 1. Introduction

Marine microorganisms are a rich source of structurally unique and bioactive metabolites. Even considering the trend of recent years that many marine natural products research efforts are directed towards microorganisms, there has been a sharp upward swing in the number of new metabolites reported from marine microorganisms [1,2]. Deep-sea organisms survive under extreme conditions such as an absence of light, low levels of oxygen, and intensely high pressures. These factors may result in the production of structurally unique secondary metabolites. Although thousands of natural products with diverse structural types have been reported from marine flora and fauna, the secondary metabolites derived from the deep-sea fungi at depths of over 1000 m below the surface are limited [3,4]. In our ongoing effort to search for structurally diverse and biologically significant metabolites from deep-sea fungi [5,6,7,8], we found that a culture broth of *Dichotomomyces cejpii* FS110, which was isolated from deep-sea sediment, showed strong growth inhibitory effects against four human tumor cell lines [9]. Bioassay-guided separation of the EtOAc extract of culture broth yielded three new diketopiperazines (**1**–**3**), along with eight known analogues. All of the isolated compounds (**1**–**11**) were evaluated for their cytotoxic activities against the SF-268, MCF-7, NCI-H460, and HepG-2 tumor cell lines and inhibitory activity against α-glucosidase. Herein, the isolation, structural elucidation, cytotoxic activities, and α-glucosidase inhibitory activities of all these isolates are described.

## 2. Results and Discussion

**Figure 1:**
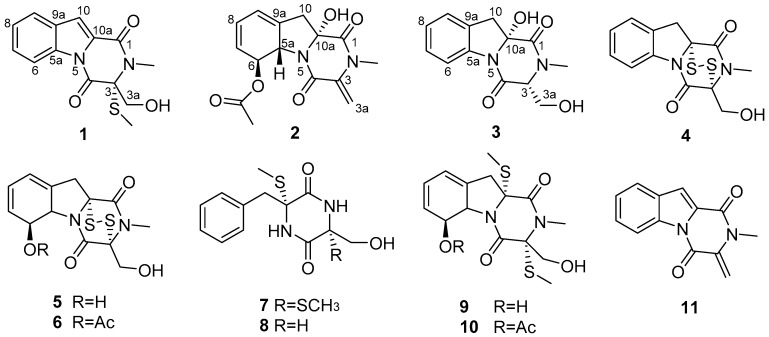
Structures of compounds **1**–**11** isolated from *Dichotomomyces cejpii* FS110.

The fermentation broth of the deep-sea-derived fungus *D. cejpii* FS110 was extracted with EtOAc and then concentrated under reduced pressure to give an extract. The EtOAc extract was subjected to various column chromatography to afford compounds **1**–**11** (Figure 1). Three new structures were identified by combination of spectroscopic analysis, single crystal X-ray diffraction, and ECD calculation, while eight known analogues were identified as 6-deoxy-5a,6-didehydrogliotoxin (**4**) [10], gliotoxin (**5**) [11,12], acetylgliotoxin (**6**) [13], bis-*N*-norgliovictin (**7**) [14], 3-benzyl-6-(hydroxymethyl)-3-(methylthio)piperazine-2,5-dione (**8**) [15], bisdethiobis(methylthio)gliotoxin (**9**) [16], 6-acetylbis(methylthio)gliotoxin (**10**) [17], and 1,2,3,4-tetrahydro-2-methyl-3-methylene-1,4-dioxopyrazino[1,2-a]indole (**11**) [18] by the comparison of their spectroscopic data (Figures S26–S47) with those in the literature.

### 2.1. Identification of New Compounds

**Figure 2:**
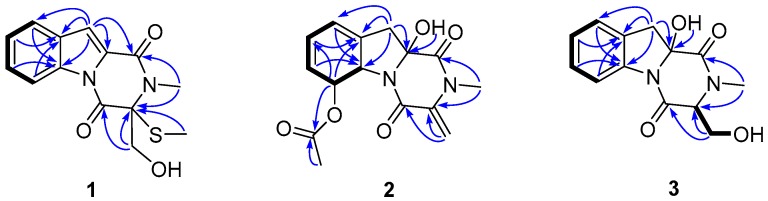
Key COSY (▬) and HMBC (→) correlations for compounds **1**–**3**.

**Figure 3:**
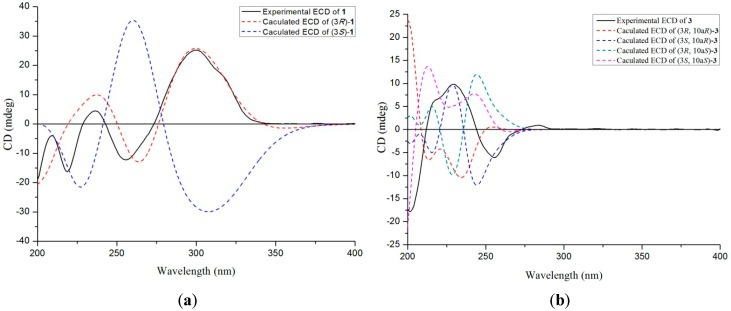
(**a**) Experimental ECD spectra of dichotocejpin A (**1**) in MeOH and calculated ECD spectra of (3*R*)-**1** and (3*S*)-**1** (**b**) Experimental ECD spectra of dichotocejpin C (**3**) in MeOH and calculated ECD spectra of (3*R,*10a*R*)-**3**, (3*S,*10a*R*)-**3**, (3*R,*10a*S*)-**3**, and (3*S,*10a*S*)-**3**. The calculated ECD spectra were computed at the B3LYP/6-31G (d) level.

Compound **1**, a pale yellow solid, had the molecular formula of C_14_H_14_N_2_O_3_S, as determined by HRESIMS (*m/z* 291.0798 [M + H]^+^, C_14_H_15_N_2_O_3_S, calcd. for 291.0803, Figure S6), corresponding to nine degrees of unsaturation. The ^1^H NMR spectrum (Figure S1) revealed the presence of two singlet methyls [δ_H_ 1.90 (3H, s) and 3.32 (3H, s)], one *O*-substituted sp^3^ methylene [δ_H_ 4.08 (1H, dd, *J* = 12.0, 7.8 Hz) and *δ*_H_ 4.46 (1H, dd, *J* = 12.0, 4.8 Hz)], an aromatic proton [δ_H_ 7.30 (1H, d, *J* = 1.0 Hz)], and a 1,2-disubstituted benzene ring [δ_H_ 7.26 (1H, ddd, *J* = 8.0, 7.2, 1.0 Hz), δ_H_ 7.42 (1H, dt, *J* = 8.0, 1.0 Hz), δ_H_ 7.47 (1H, ddd, *J* = 8.3, 7.2, 1.0 Hz) and δ_H_ 8.44 (1H, dt, *J* = 8.3, 1.0 Hz)]. The ^13^C NMR, in combination with DEPT experiments (Figure S2), resolved 14 carbon resonances attributed to two amide carbonyls (δ_C_ 158.0, 163.5), three sp^2^ quaternary carbons, five sp^2^ methines, one sp^3^ quaternary carbon, one sp^3^ methylene, and two methyls (Table 1). The 1D NMR data of **1** showed resonances characteristic of a diketopiperazine framework similar to that of neosartin B [19], with the exception of a thiomethyl at δ_C_ 12.2 replacing a methoxyl of the latter, and this assignment was supported by the HMBC correlations (Figure 2) from the thiomethyl (δ_H_ 1.90) to the severely upfiled-shifted C-3 (δ_C_ 76.7 in **1** and 93.6 in neosartin B). Detailed 2D analyses (HSQC, ^1^H–^1^H COSY, and HMBC, Figures S3–S5) supported the planar structure of **1** as depicted (Figure 2). HMBC correlations from the *N*-methyl (δ_H_ 3.32) to an amide carbonyl (δ_C_ 158.0, C-1) and a sp^3^ quaternary carbon (δ_C_ 76.7, C-3) and from the *O*-substituted sp^3^ methylene (δ_H_ 4.46 and 4.08, H_2_-3a) to another amide carbonyl (δ_C_ 163.5, C-4), and the sp^3^ quaternary carbon revealed the diketopiperazine framework. COSY correlations of H-6/H-7/H-8/H-9 and HMBC correlations from H-10 (δ_H_ 7.30) to C-9 (δ_C_ 122.8), C-9a (δ_C_ 134.9), C-5a (δ_C_ 129.1), C-10a (δ_C_ 127.4), and C-1 (δ_C_ 158.0) and from H-6 (δ_H_ 8.44) to C-9a (δ_C_ 134.9) suggested the remaining fragments. The absolute structure of **1** was deduced by comparison of the experimental and simulated electronic circular dichroism (ECD) spectra generated by time-dependent density functional theory (TDDFT) calculations (B3LYP/6-31G (d)) with Gaussian 09 [20]. The experimental ECD spectrum of **1** showed an ECD curve with Cotton effects around 300 (+), 256 (−) and 237 (+) nm, respectively (Figure 3a), which was in good accordance with the calculated ECD spectrum for (3*R*)-**1**, indicating that **1** had a 3*R*-configuration. Compound **1** was given the trivial name dichotocejpin A.

Compound **2**, a colorless crystal, had the molecular formula of C_15_H_16_N_2_O_5_, as determined by HRESIMS (*m/z* 327.0953 [M + Na]^+^, C_1__5_H_1__6_N_2_O_5_Na; calcd. for 327.0957, Figure S15), corresponding to nine degrees of unsaturation. The ^1^H NMR spectrum (Figure S9) revealed the presence of two singlet methyls [δ_H_ 2.02 (3H, s) and 3.11 (3H, s)], one sp^3^ methylene [δ_H_ 2.75 (1H, d, *J* = 15.3 Hz) and 2.88 (1H, d, *J* = 15.3 Hz)], two *O*- or *N*-substituted sp^3^ methines [δ_H_ 4.98 (1H, d, *J* = 14.0 Hz) and 5.75 (1H, d, *J* = 14.0 Hz)], one terminal double bond methylene [δ_H_ 5.02 (1H, d, *J* = 1.1 Hz) and 5.48 (1H, d, *J* = 1.1 Hz)], three sp^2^ methines [δ_H_ 5.54 (1H, d, *J* = 9.6 Hz), 5.94 (1H, m) and 5.99 (1H, m)], and a deshielded hydroxyl group [δ_H_ 7.14 (1H, s)]. The ^13^C NMR, in combination with DEPT experiments (Figure S10), resolved 15 carbon resonances attributed to three ester or peptide carbonyl carbons, two sp^2^ quaternary carbons, three sp^2^ methines, one sp^2^ methylene, one sp^3^ methylene, and two methyls. The above-mentioned information was quite similar to the diketopiperazine bis(dethio)-10a-methylthio-3a-deoxy-3,3a-didehydrogliotoxin [10], except for the presence of a acetoxyl group and the absence of a single methyl in **2**, which was further confirmed by ^1^H–^1^H COSY correlations of C-6/C-7/C-8/C-9 (Figure 2) and HMBC correlations from H-6 (δ_H_ 5.75) to the acetyl (δ_C_ 169.9) (Figures S11–S14).

**Figure 4:**
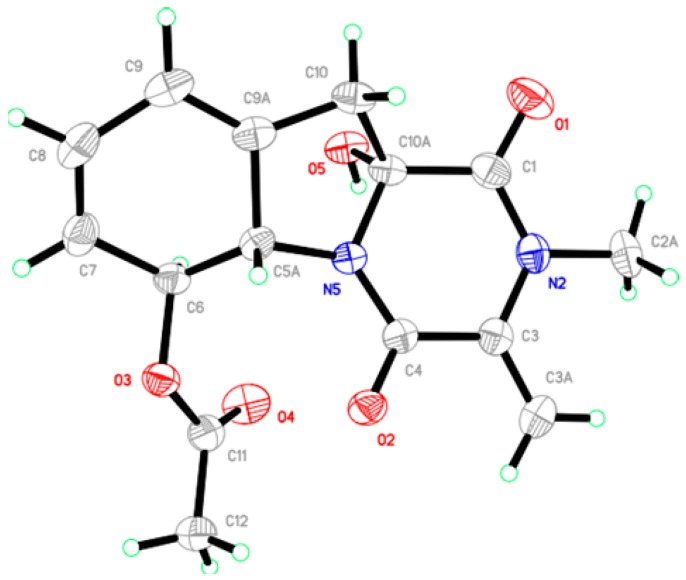
ORTEP diagram of compound **2**.

As molecules possessing quaternary chiral center widely exist in nature, it has proven challenging via conventional spectroscopic methods to assign which antipodal series compound **2** belongs to. We thus resorted to a single-crystal X-ray diffraction experiment (Figure 4) using Cu Kα radiation (λ = 1.54184 Å). The structure including the absolute configuration of **2** was confirmed, and C-5a, C-6, and C-10a were assigned to *S*, *S*, *R* (absolute structure parameter: −0.12). Thus, the structure of **2** was established and given the trivial name dichotocejpin B.

Compound **3**, a white solid, had the molecular formula of C_13_H_14_N_2_O_4_, as determined by HRESIMS (*m/z* 261.0888 [M − H]^−^, C_13_H_13_N_2_O_4_, calcd. for 261.0875, Figure S23), corresponding to eight degrees of unsaturation. The 1D NMR data of **3** (Figures S18 and S19) showed that it possessed the same diketopiperazine skeleton as **1**, and was quite similar to the known compound 6-deoxy-5a,6-didehydrogliotoxin [10]. The main differences between them were the presence of a triplet hydrogen and the downfield-shifted C-3 (δ_C_ 66.1 in **3** and δ_C_ 74.0 in 6-deoxy-5a,6-didehydrogliotoxin) and C-10a in **3** (δ_C_ 88.0 in **2** and δ_C_ 74.0 in 6-deoxy-5a,6-didehydrogliotoxin). The molecular weight of **3** was 46 amu, less than that of 6-deoxy-5a,6-didehydrogliotoxin, indicating the absence of the disulphide bond between C-3 and C-10a. HMBC correlations from OH-10a to C-10, C-10a, and C-1 revealed **3** was a 10a-OH derivative of 6-deoxy-5a,6-didehydrogliotoxin. This was supported by detailed 2D NMR spectra (Figure 2, Figures S20–S22) analyses.

The absolute structure of **3** was also deduced by comparison of the experimental and calculated ECD spectra generated by TDDFT calculations. As illustrated in Figure 3b, the experimentally acquired CD spectrum for **3** agreed well with the ECD curve computed for (3*S*, 10a*R*)-**3**. Thus, the most likely absolute configuration of **3** was established as 3*S*, 10a*R*. Compound **3** was given the trivial name dichotocejpin C.

### 2.2. Cytotoxicity Assay

The in vitro cytotoxicities of compounds **1**–**11** were evaluated against the SF-268, MCF-7, NCI-H460, and HepG-2 tumor cell lines by the Sulforhodamine B (SRB) method. Compounds **4**–**6**, which have a disulfide bond, exhibited the most potent inhibitory activities against the four tumor cell lines with IC_50_ values in the range of 0.08–1.52 μM (Table 2). Compounds **10** and **11** also showed significant inhibitory activities, whereas compounds **1**, **2**, **3**, and **7**–**9** were inactive or weak against these tumor cell lines.

### 2.3. α-Glucosidase Inhibitory Activity Assay

All compounds were evaluated in vitro for *α*-glucosidase inhibitory activities. Compound **1** showed excellent inhibitory activity against *α*-glucosidase (IC_50_ = 138 μM), being stronger than the positive control acarbose (an oral antidiabetic agent, IC_50_ = 463 μM).

## 3. Materials and Methods

### 3.1. General Experimental Procedures

Optical rotations were measured using an Anton Paar MCP-500 (Anton Paar, Graz, Austria). Circular dichroism (CD) measurements were carried out under N_2_ gas on a Jasco 820 spectropolarimeter (Jasco Corporation, Kyoto, Japan). IR spectra were recorded on a Shimadzu IR Affinity-1 spectrophotometer (Shimadzu Corporation, Kyoto, Japan). UV spectra were measured on a Shimadzu UV-2600 spectrophotometer (Shimadzu Corporation, Kyoto, Japan). NMR spectra were recorded on a Bruker Avance-500 spectrometer (Bruker Corporation, Fremont, CA, USA) and referenced to the signals of tetramethylsilane as an internal standard. HRESIMS was measured on a Thermo MAT95XP high-resolution mass spectrometer (Thermo Fisher Scientific, Bremen, Germany) and ESIMS was measured on an Agilent Technologies 1290-6430A Triple Quad LC/MS (Agilent Technologies Inc., Santa Clara, CA, USA). A Shimadzu LC-20 AT (Shimadzu Corporation, Kyoto, Japan) equipped with an SPD-M20A PDA detector (Shimadzu Corporation, Kyoto, Japan) was used for HPLC, a YMC-pack ODS-A column (250 × 10 mm, 5 μm, 12 nm) was used for semi-preparative HPLC separation, and a YMC-pack SIL (250 × 20 mm, 5 μm, 12 nm, YMC CO., Ltd., Kyoto, Japan) and a YMC-pack ODS-A column (250 × 20 mm, 5 μm, 12 nm, YMC CO., Ltd., Kyoto, Japan) were used for preparative HPLC separation. Column chromatography: commercial silica gel (SiO_2_; 200–300 mesh; Qingdao Marine Chemical Plant, Qingdao, China) and Sephadex LH-20 gel (Amersham Biosciences, Uppsala, Sweden). All solvents were of analytical grade (Guangzhou Chemical Regents Company, Ltd., Guangzhou, China).

### 3.2. Fungal Material and Identification

The fungal strain FS110 was isolated from a deep-sea sedimental sample in the South China Sea (19°0.368′ N, 117°58.233′ E; depth 3941 m). The isolate was identified as *Dichotomomyces cejpii* FS110 based on ITS rDNA sequence analysis [9]. The sequence of the ITS region of the strain FS110 has been submitted to GenBank (Accession No. KF706672). By using BLAST (nucleotide sequence comparison program) to search the GenBank database, FS110 has 99.8% similarity with *Dichotomomyces cejpii* NRRL 26980 (Accession No. EF669956). The strain was preserved at the Guangdong Provincial Key Laboratory of Microbial Culture Collection and Application, Guangdong Institute of Microbiology. Working stocks were prepared on potato dextrose agar (PDA) slants and stored at 4 °C.

### 3.3. Fermentation, Extraction, and Isolation

A well-grown slant culture of *D. cejpii* FS110 was used for preparation of the seed culture. Three pieces (0.5 × 0.5 cm^2^) of mycelial agar plugs of this strain were inoculated into 250 mL of PD medium (potato 200 g/L, glucose 20 g/L, KH_2_PO_4_ 3 g/L, MgSO_4_∙7H_2_O 1.5 g/L, vitamin B_1_ 10 mg/L, sea salt 15 g/L) in a 500 mL Erlenmeyer flask, and incubated for 2 days in a rotary shaker (200 r/m) at 28 °C. The seed cultures (10%) were then aseptically transferred into 500 mL of PD medium in 1000 mL Erlenmeyer flasks and shaken (120 r/m) at 28 °C for 7 days. A total of 100 L of fermentation broth was filtered to give the broth and mycelia. The broth was partitioned sequentially with EtOAc (4 × 25 L) to yield a dark brown oily residue (40.0 g), which was subjected to column chromatography on silica gel eluted with petroleum ether/EtOAc in linear gradient (30:1→1:1) and followed by CHCl_3_/MeOH in linear gradient (10:1→0:1) to give 26 fractions (F1–F26). F2 was washed with MeOH to get **4** (4.8 mg). F6 was separated on a preparative reversed-phase (RP) HPLC system equipped with a YMC column (MeOH/H_2_O, 50:50, 10 mL/min) to yield **1** (1.4 mg, *t_R_* = 17 min) and **6** (1.0 g, *t_R_* = 25 min). F10 was separated by column chromatography on Sephadex LH-20 (CHCl_3_/MeOH, 1:1, *v*/*v*) to yield **5** (2.0 mg). F11 was subjected to a Sephadex LH-20 column eluting with CHCl_3_/MeOH (1:1), then further purified by column chromatography on silica gel eluted with petroleum ether/EtOAc (5:1) to yield **7** (4.8 mg). F12 was separated by column chromatography on Sephadex LH-20 (CHCl_3_/MeOH, 1:1, *v*/*v*), then further separated by semi-preparative HPLC on a C-18 column (YMC*GEL ODS-A, 120A S-5 μm, 250 × 10 mm, MeOH/H_2_O, 40:60, 2 mL/min) to yield **2** (5.0 mg, *t*_R_ = 16.0 min), **9** (75.0 mg, *t*_R_ = 25.0 min), and **10** (75.0 mg, *t*_R_ = 31.0 min). F13 was separated by column chromatography on Sephadex LH-20 (CHCl_3_/MeOH, 1:1, *v*/*v*) and then further purified by semi-preparative HPLC on a C-18 column (YMC*GEL ODS-A, 120A S-5 μm, 250 × 10 mm, MeOH/H_2_O, 40:60, 2 mL/min) to yield **11** (3.3 mg, *t*_R_ = 18 min). F17 was separated by preparative HPLC on a C-18 column (YMC*GEL ODS-A, 120A S-5 μm, 250 × 20 mm, MeOH/H_2_O, 50:50, 10 mL/min) to give 5 fractions (F17.1–F17.5). F17.2 was further purification by semi-preparative HPLC on a C-18 column (YMC*GEL ODS-A, 120A S-5 μm, 250 × 10 mm, MeCN/H_2_O, 30:70, 2 mL/min) to give **8** (50.2 mg, *t*_R_ = 34 min). F17.3 was further purification by semi-preparative HPLC on a C-18 column (YMC*GEL ODS-A, 120A S-5 μm, 250 × 10 mm, MeCN/H_2_O, 20:80, 2 mL/min) to give **3** (9.0 mg, *t*_R_ = 19 min).

Dichotocejpin A (**1**): pale yellow solid; [α]${}_{D}^{25}$ +126.2 (*c* 0.1, MeOH); CD (MeOH, *c* 0.25 mg/mL) 209, 219, 237, 256 and 300 nm (∆ε −1.19, −4.93, +1.35, −3.71 and +7.63); UV (MeOH) λ_max_ (log ε) 207 (4.37), 244 (4.34), 296 (4.15) nm (Figure S8); IR ν_max_ 3372, 2955, 2922, 2853, 1709, 1636, 1587, 1429, 1391, 1358, 1259, 1026 cm^−1^ (Figure S7); ^1^H and ^13^C NMR data, see Table 1; HRESIMS *m/z* 291.0798 ([M + H]^+^, calcd for 291.0803).

Dichotocejpin B (**2**): colorless crystal; [α]${}_{D}^{25}$ +93.5 (*c* 0.1, MeOH); CD (MeOH, *c* 0.25 mg/mL) 217, 233 and 268 nm (∆*ε* −1.91, +1.00 and −4.68); UV (MeOH) λ_max_ (log ε) 205 (4.55), 232 (4.59) nm (Figure S17); IR ν_max_ 3273, 2958, 2924, 2855, 1732, 1682, 1612, 1367, 1258, 1026 cm^−1^ (Figure S16); ^1^H and ^13^C NMR data, see Table 1; HRESIMS *m/z* 327.0953 ([M + Na]^+^, calcd for 327.0957).

Dichotocejpin C (**3**): white solid; [α]${}_{D}^{25}$ +44.4 (*c* 0.1, MeOH); CD (MeOH, *c* 0.25 mg/mL) 230, 256 and 284 nm (∆*ε* +2.98, −1.85 and +0.29); UV (MeOH) λ_max_ (log ε) 206 (4.26), 246 (3.88), 277 (3.48), 284 (3.46) nm (Figure S25); IR ν_max_ 3294, 2941, 1672, 1643, 1603, 1487, 1431, 1400, 1078, 1056 cm^−1^ (Figure S24); ^1^H and ^13^C NMR data, see Table 1; HRESIMS *m/z* 261.0888 ([M − H]^−^, calcd for 261.0875).

### 3.4. Quantum-Chemical ECD Calculation

The quantum-chemical ECD calculation methods were used to establish the absolute configurations of compounds **1** and **3**. The compounds were charged using the Gasteiger–Hückel method and the preliminary random conformational search was performed with the SYBYL 8.0 software package using the MMFF94 molecular mechanics force field. The geometry optimizations were then performed by using DFT at the B3LYP/6-31G (d) level as implemented in the Gaussian 09 program package. The stable conformers obtained were subsequently submitted to ECD calculations by TDDFT calculations (B3LYP/6-31G (d)) with Gaussian 09.

### 3.5. Crystallographic Data for **2**

X-ray crystal data for dichotocejpin B (**2**). C_15_H_16_N_2_O_5_, M = 304.30 g/mol, orthorhombic, crystal size 0.400 × 0.320 × 0.230 mm^3^, space group *P*2_1_2_1_2_1_, a = 8.58255(15) Å, b = 10.5549(2) Å, c = 16.0203 (3) Å, α = β = γ = 90°, V = 1451.24(5) Å^3^, Z = 4, D_calcd_ = 1.393 mg/m^3^, F(000) = 640. μ = 0.889 mm^−1^, Cu Kα radiation, λ = 1.54184 Å, T = 150 (2) K, 5.018° ≤ 2θ ≤ 66.890°, 9943 reflections collected, 2531 unique (Rint = 0.0291). Final GooF = 1.123, R^1^ = 0.0310, wR^2^ = 0.0748 based on 2531 reflections with I > 2σ(I) (refinement on *F*^2^), 424 parameters, 42 restraint. Lp and absorption corrections applied, m = 0.091 mm_1. Flack parameter = −0.12 (9). Crystallographic data for **1** have been deposited with the Cambridge Crystallographic Data Centre as supplementary publication No. CCDC 1491672. Copies of the data can be obtained, free of charge, on application to the CCDC, 12 Union Road, Cambridge CB2 1EZ, UK (fax, +44-(0)-1223-336033; e-mail, deposit@ccdc.cam.ac.uk).

### 3.6. Cytotoxicity Assay

Cytotoxicities of compounds (**1**–**11**) were assayed against four tumor cell lines SF-268, MCF-7, NCI-H460, and HepG-2. Assays were performed by the SRB method [21]. 

### 3.7. α-Glucosidase Inhibitory Activity Assay

An assay of α-glucosidase inhibitory activity was performed as previously described [22]. 

## 4. Conclusions

In this study, eleven diketopiperazines, including three new ones, were isolated from the deep-sea-derived fungus *Dichotomomyces cejpii*. All the chemical structures, including absolute configurations, were established. Compounds **4**–**6**, **10**–**11** exhibited significant cytotoxic activities against SF-268, MCF-7, NCI-H460, and HepG-2 tumor cell lines. By comparing the structures of these compounds, compounds **4**–**6**, with a disulfide bridge, showed stronger cytotoxicities, which is accordance with the investigations reported in the literature [3,10,19]. Compound **1** is the first case of thio-diketopiperazines with excellent inhibitory activity against α-glucosidase, which might be useful for further developing α-glucosidase inhibitor and antidiabetic agents.

## Figures and Tables

**Table 1:** ^1^H (500 MHz) and ^13^C (125 MHz) NMR data for compounds **1**–**3** (δ in ppm, *J* in Hz).

Position	1 ^a^		2 ^b^		3 ^b^	
	δ_H_, mult. (*J* in Hz)	δ_C_, Type	δ_H_, mult. (*J* in Hz)	δ_C_, Type	δ_H_, mult. (*J* in Hz)	δ_C_, Type
1		158.0, C		164.2, C		165.2, C
3		76.7, C		138.4, C	4.29, t (3.6)	66.1, CH
3a	4.46, dd (12.0, 7.8) 4.08, dd (12.0, 4.8)	64.2, CH_2_	5.48, d (1.1) 5.02, d (1.1)	102.1, CH_2_	3.96, m	60.5, CH_2_
4		163.5, C		157.0, C		164.6, C
5a		129.1, C	4.98, d (14.0)	63.0, CH		128.8, C
6	8.44, dt (8.3, 1.0)	116.9, CH	5.75, d (14.0)	75.3, CH	7.97, d (7.7)	115.5, CH
7	7.47, ddd (8.3, 7.2, 1.0)	128.3, CH	5.54, d (9.6)	127.4, CH	7.26, t (7.7)	127.3, CH
8	7.26, ddd (8.0, 7.2, 1.0)	125.9, CH	5.99, m	125.4, CH	7.13, td (7.7, 1.1)	124.7, CH
9	7.42, dt (8.0, 1.0)	122.8, CH	5.94, m	118.5, CH	7.35, d (7.7)	125.2, CH
9a		134.9, C		135.9, C		140.0, C
10	7.30, d (1.0)	115.1, CH	2.88, d (15.3) 2.75, d (15.3)	41.7, CH_2_	3.53, d (17.2) 3.14, d (17.2)	39.7, CH_2_
10a		127.4, C		88.7, C		88.0, C
N-CH_3_	3.32, s	28.2, CH_3_	3.11, s	29.6, CH_3_	2.98, s	32.2, CH_3_
S-CH_3_	1.90, s	12.2, CH_3_				
OH-3a	3.39, t (7.1)				6.53, brs	
OH-10a			7.14, s		6.88, brs	
OAc			2.02, s	169.9, C 21.3, CH_3_		

^a^ Recorded in CDCl_3_; ^b^ Recorded in DMSO-*d*_6_.

**Table 2:** IC_50_ values of compounds **1**–**11** against four tumor cell lines and α-glucosidase.

Compounds	IC_50_ (μM) ^a^				
	SF-268	MCF-7	NCI-H460	HepG-2	α-glucosidase
**1**	35.7 ± 2.1	29.5 ± 2.3	>100	28.9 ± 3.0	138 ± 6.7
**2**	>100	>100	>100	>100	>500
**3**	>100	>100	>100	>100	>500
**4**	1.35 ± 0.05	0.68 ± 0.02	1.27 ± 0.04	1.52 ± 0.03	>500
**5**	0.24 ± 0.10	0.08 ± 0.0	0.24 ± 0.01	0.21 ± 0.01	>500
**6**	0.25 ± 0.03	0.22 ± 0.04	0.32 ± 0.02	0.49 ± 0.07	>500
**7**	>100	>100	>100	>100	>500
**8**	>100	>100	>100	>100	>500
**9**	>100	>100	>100	>100	>500
**10**	34.0 ± 3.6	3.1 ± 0.10	5.4 ± 0.60	7.0 ± 0.17	>500
**11**	3.3 ± 0.28	4.67 ± 1.4	12.3 ± 0.24	2.29 ± 0.30	>500
positive control	2.37 ± 0.35 ^b^	3.09 ± 0.27 ^b^	2.43 ± 0.15 ^b^	1.39 ± 0.18 ^b^	463 ± 35 ^c^

^a^ $\bar{x}\pm s,n=3$; ^b^ Positive control was cisplatin; ^c^ Positive control was acarbose.

## Supplementary Materials

The HRESIMS, IR, UV, and 1D and 2D-NMR spectra of compounds **1**–**3** (Figures S1–S25), ESIMS and 1D-NMR data of **4**–**11** (Figures S26–S47), can be accessed at: http://www.mdpi.com/1660-3397/14/9/164/s1.
