# Supplementary material for: Dichotocejpins A–C: New Diketopiperazines from a Deep-Sea-Derived Fungus Dichotomomyces cejpii FS110
Source: Mar Drugs. 2016 Sep 9;14(9):164. doi: 10.3390/md14090164 (PMC5039535; doi:10.3390/md14090164)
Supplement: Supplementary file 1 [file marinedrugs-14-00164-s001.pdf]

Supplementary Materials: Dichotocejpins A–C: New Diketopiperazines from a Deep-Sea-Derived Fungus *Dichotomomyces cejpii* FS110

Zhen Fan, Zhang-Hua Sun, Zhong Liu, Yu-Chan Chen, Hong-Xin Liu, Hao-Hua Li and Wei-Min Zhang

List of Supporting Information

Figure S1. ^{1}H NMR spectrum of dichotocejpin A (**1**) in $CDCl_3$.
Figure S2. ^{13}C NMR and DEPT135 spectra of dichotocejpin A (**1**) in $CDCl_3$.
Figure S3. HSQC spectrum of dichotocejpin A (**1**) in $CDCl_3$.
Figure S4. ^{1}H–^{1}H COSY spectrum of dichotocejpin A (**1**) in $CDCl_3$.
Figure S5. HMBC spectrum of dichotocejpin A (**1**) in $CDCl_3$.
Figure S6. HRESIMS spectrum of dichotocejpin A (**1**).
Figure S7. IR spectrum of dichotocejpin A (**1**).
Figure S8. UV spectrum of dichotocejpin A (**1**).
Figure S9. ^{1}H NMR spectrum of dichotocejpin B (**2**) in DMSO-d_6.
Figure S10. ^{13}C NMR and DEPT135 spectra of dichotocejpin B (**2**) in DMSO-d_6.
Figure S11. HSQC spectrum of dichotocejpin B (**2**) in DMSO-d_6.
Figure S12. ^{1}H–^{1}H COSY spectrum of dichotocejpin B (**2**) in DMSO-d_6.
Figure S13. HMBC spectrum of dichotocejpin B (**2**) in DMSO-d_6.
Figure S14. NOESY spectrum of dichotocejpin B (**2**) in DMSO-d_6.
Figure S15. HRESIMS spectrum of dichotocejpin B (**2**).
Figure S16. IR spectrum of dichotocejpin A (**2**).
Figure S17. UV spectrum of dichotocejpin A (**2**).
Figure S18. ^{1}H NMR spectrum of dichotocejpin C (**3**) in DMSO-d_6.
Figure S19. ^{13}C NMR spectrum of dichotocejpin C (**3**) in DMSO-d_6.
Figure S20. HSQC spectrum of dichotocejpin C (**3**) in DMSO-d_6.
Figure S21. ^{1}H–^{1}H COSY spectrum of dichotocejpin C (**3**) in DMSO-d_6.
Figure S22. HMBC spectrum of dichotocejpin C (**3**) in DMSO-d_6.
Figure S23. HRESIMS spectrum of dichotocejpin C (**3**) in DMSO-d_6.
Figure S24. IR spectrum of dichotocejpin A (**3**).
Figure S25. UV spectrum of dichotocejpin A (**3**).
Figure S26. ^{1}H NMR spectrum of compound **4** in CD_3OD.
Figure S27. ESI-MS spectrum of compound **4**.
Figure S28. ^{1}H NMR spectrum of compound **5** in CD_3OD.
Figure S29. ^{13}C NMR spectrum of compound **5** in CD_3OD.
Figure S30. ^{1}H NMR spectrum of compound **6** in $CDCl_3$.
Figure S31. ^{13}C NMR spectrum of compound **6** in $CDCl_3$.
Figure S32. ESI-MS spectrum of compound **6**.
Figure S33. ^{1}H NMR spectrum of compound **7** in DMSO-d_6.
Figure S34. ^{13}C NMR spectrum of compound **7** in DMSO-d_6.
Figure S35. ESI-MS spectrum of compound **7**.
Figure S36. ^{1}H NMR spectrum of compound **8** in DMSO-d_6.
Figure S37. ^{13}C NMR spectrum of compound **8** in DMSO-d_6.
Figure S38. ESI-MS spectrum of compound **8**.
Figure S39. ^{1}H NMR spectrum of compound **9** in CD_3OD.
Figure S40. ^{13}C NMR spectrum of compound **9** in CD_3OD.
Figure S41. ESI-MS spectrum of compound **9**.
Figure S42. ^{1}H NMR spectrum of compound **10** in CD_3OD.
Figure S43. ^{13}C NMR spectrum of compound **10** in CD_3OD.

Figure S44. ESI-MS spectrum of compound **10**.
Figure S45. ^{1}H NMR spectrum of compound **11** in DMSO-d_6.
Figure S46. ^{13}C NMR spectrum of compound **11** in $CDCl_3$.
Figure S47. ESI-MS spectrum of compound **11**.

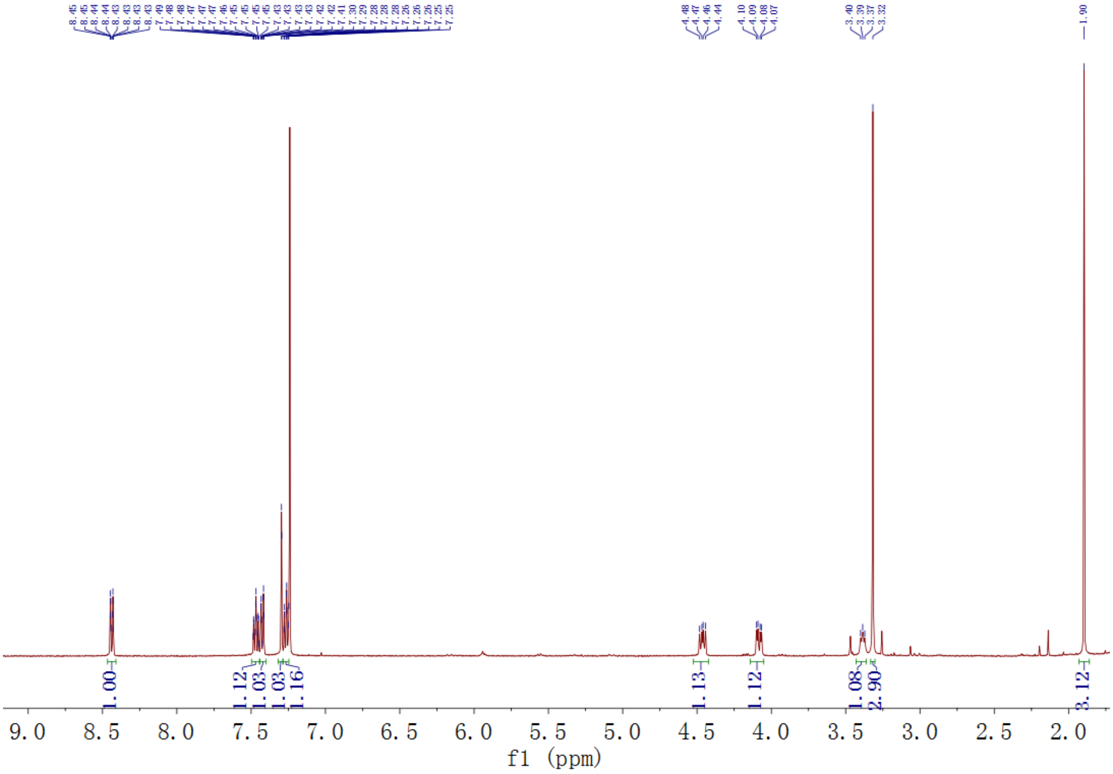

Figure S1. ^{1}H NMR spectrum of dichotocejpin A (**1**) in $CDCl_3$.

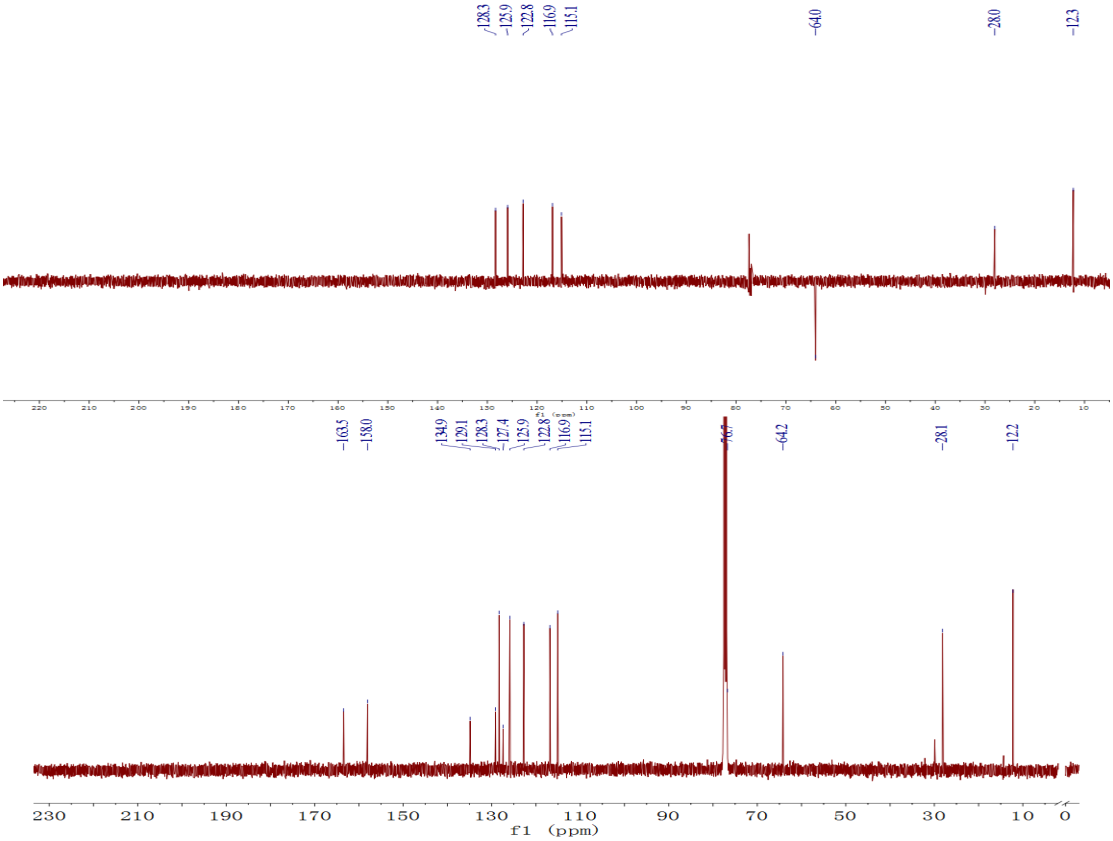

Figure S2. ^{13}C NMR and DEPT135 spectrum of dichotocejpin A (**1**) in $CDCl_3$.

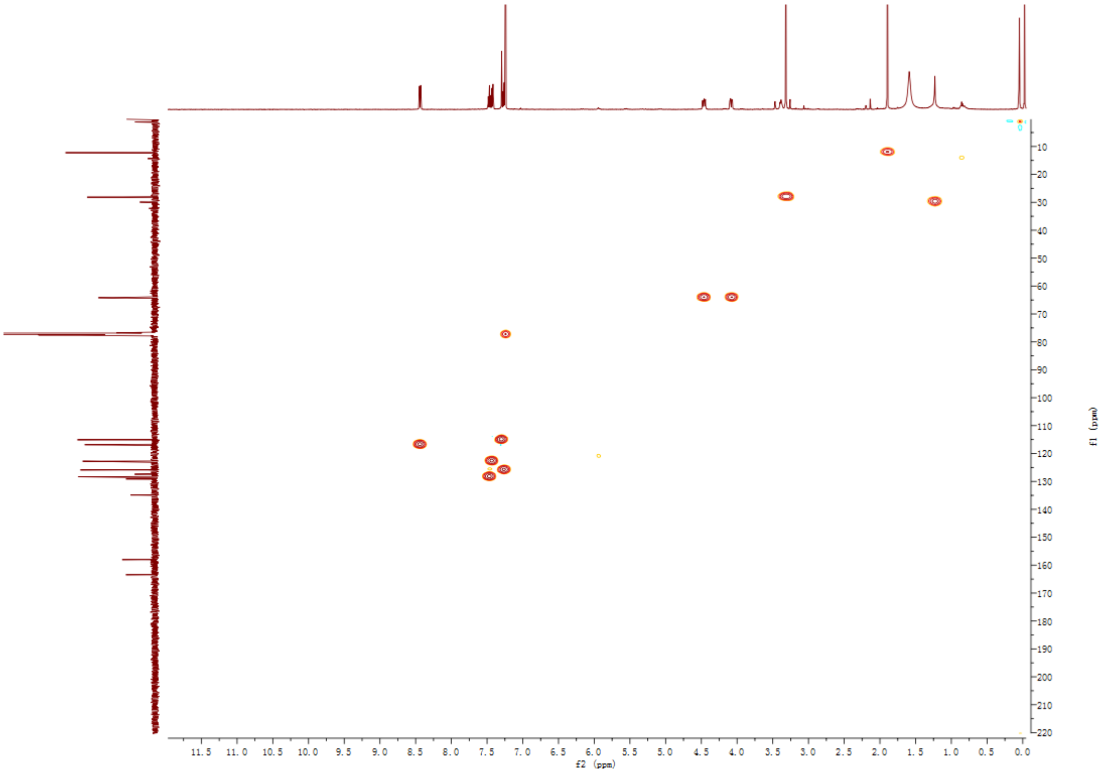

Figure S3. HSQC spectrum of dichotocejpin A (**1**) in $CDCl_3$.

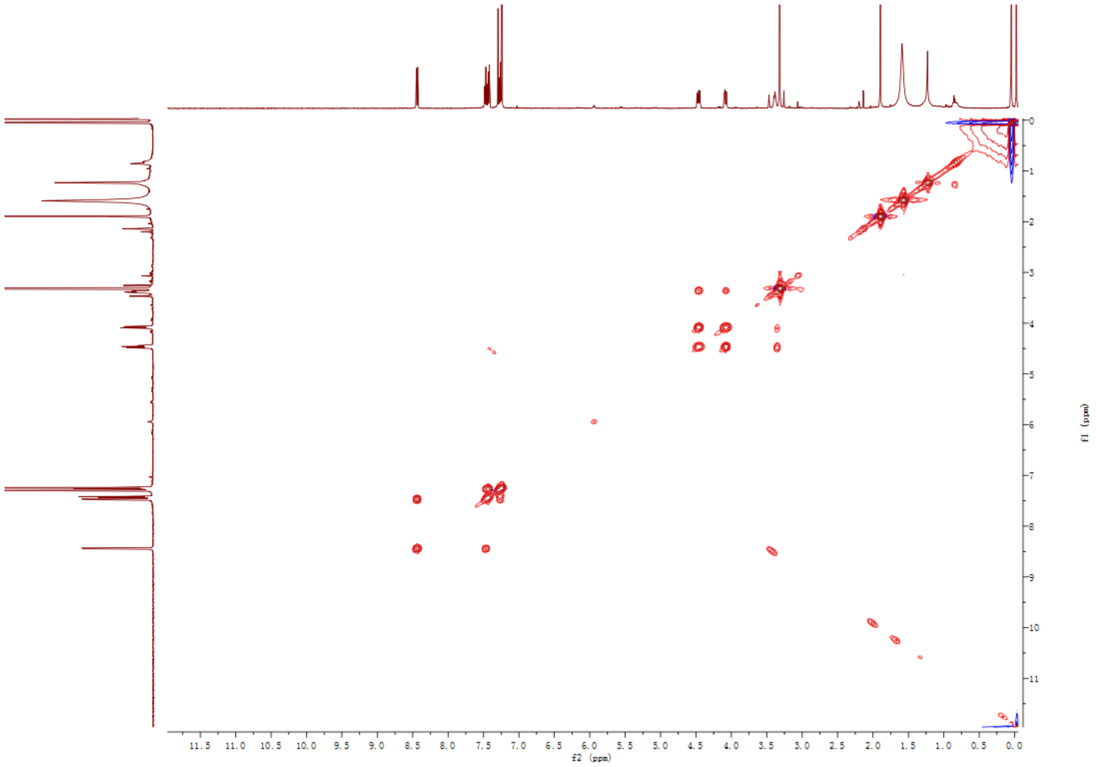

Figure S4. ^{1}H–^{1}H COSY spectrum of dichotocejpin A (**1**) in $CDCl_3$.

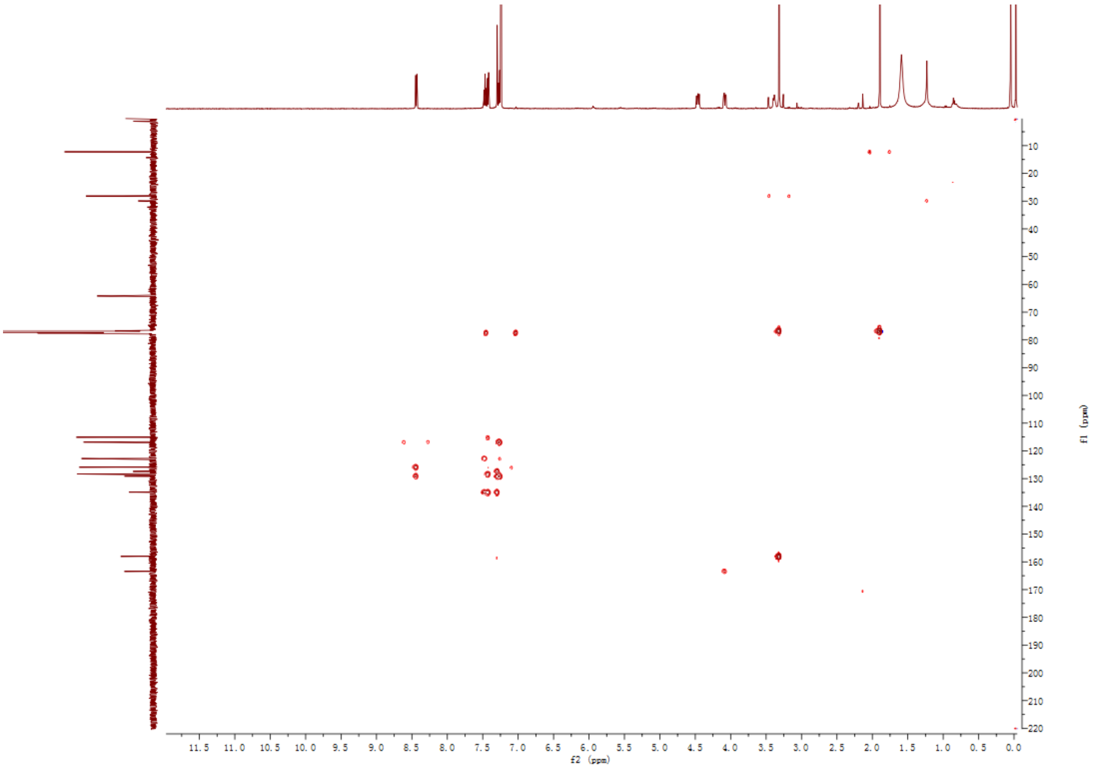

Figure S5. HMBC spectrum of dichotocejpin A (**1**) in $CDCl_3$.

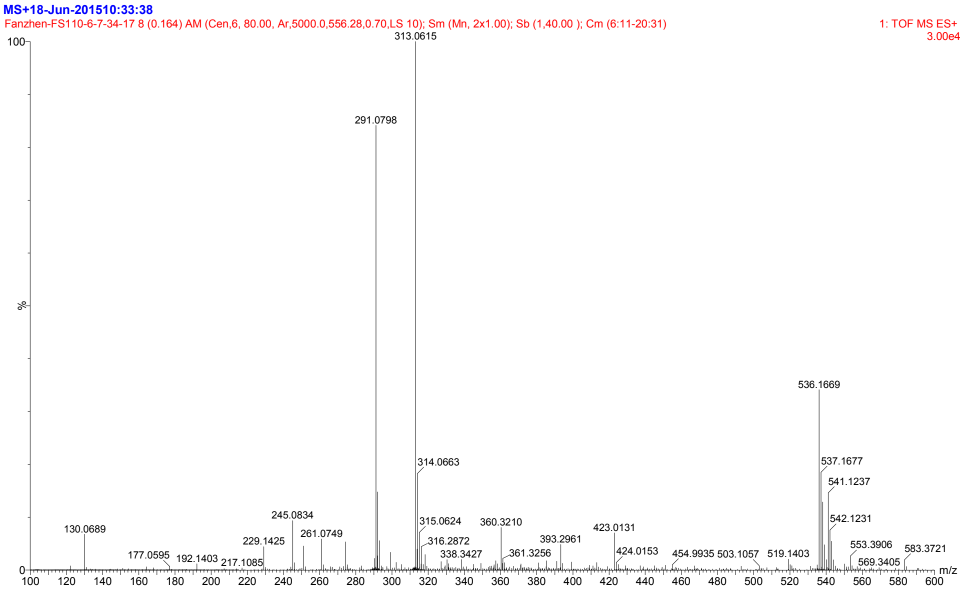

Figure S6. HRESIMS spectrum of dichotocejpin A (**1**).

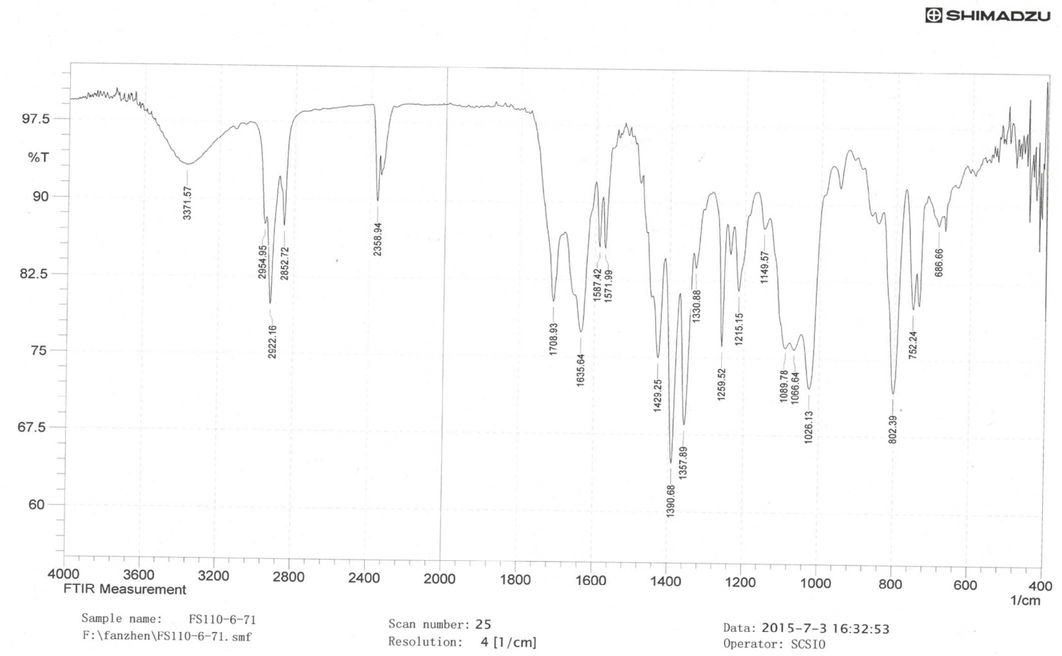

Figure S7. IR spectrum of dichotocejpin A (**1**).

光谱峰值检测报告

2015-07-03 14:56:30

数据集: FS110-6-7-2 - RawData

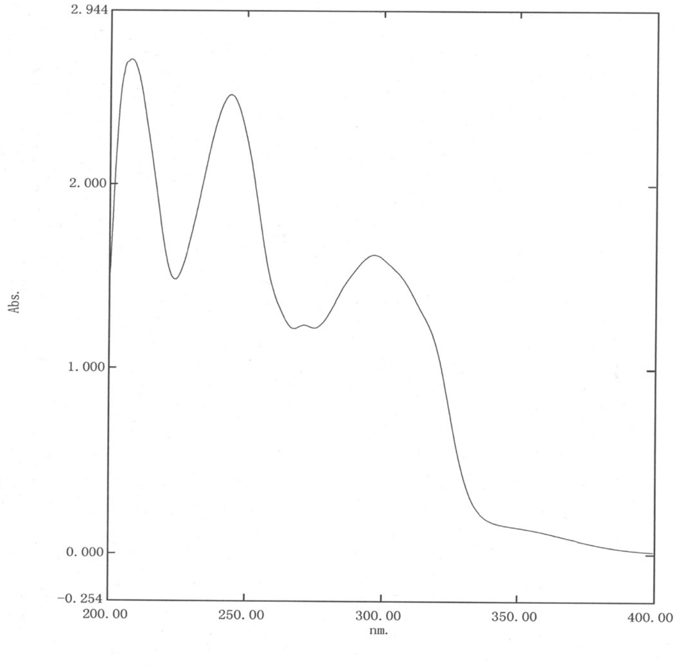

[测定属性]
波长范围 (nm.): 200.00 到 400.00
扫描速度: 中速
采样间隔: 0.2
自动采样间隔: 启用
扫描模式: 单个

[仪器属性]
仪器类型: UV-2600 系列
测定方式: 吸收值
狭缝宽: 2.0
积分时间: 0.1 秒.
光源转换波长: 323.0 nm
检测器单元: 直接
S/R 转换: 标准
阶梯校正: OFF

[附件属性]
附件: 无

[数据处理参数]
阈值: 0.0100000
点: 4
内插: 停用
平均: 停用

[样品准备属性]
重量:
体积:
稀释:
光程长:
附加信息:

No.	P/V	波长(nm)	吸收值	描述
1	↑	296.60	1.620	
2	↑	271.40	1.237	
3	↑	244.40	2.488	
4	↑	207.80	2.677	
5	↓	275.00	1.222	
6	↓	267.60	1.219	
7	↓	224.20	1.484	

Figure S8. UV spectrum of dichotocejpin A (**1**).

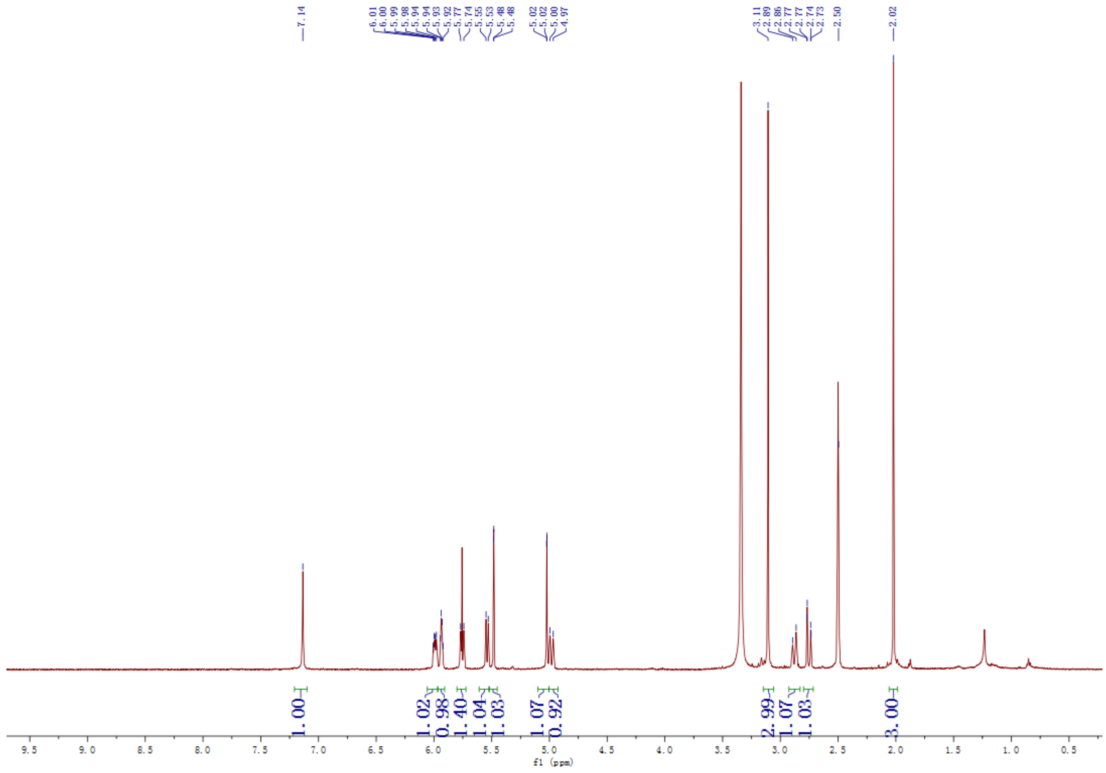

Figure S9. ^{1}H NMR spectrum of dichotocejpin B (**2**) in DMSO-d_6.

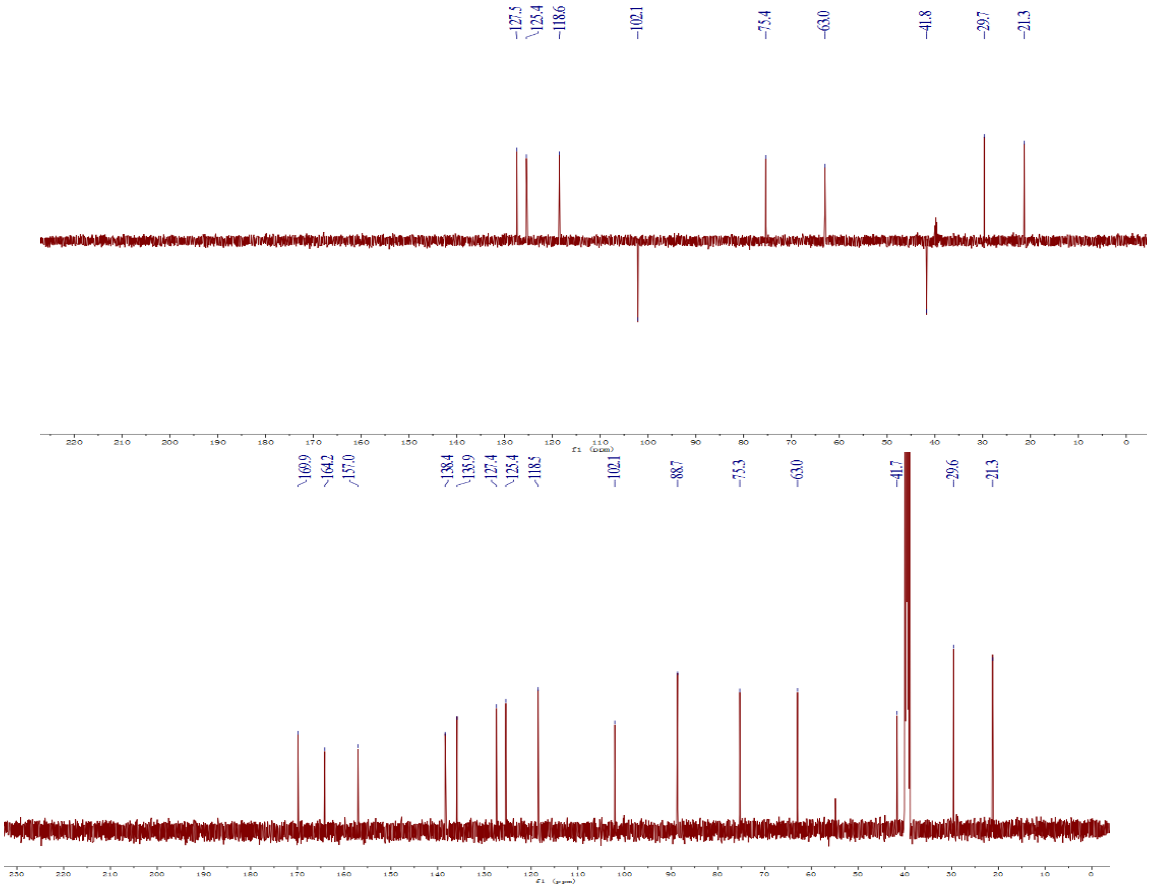

Figure S10. ^{13}C NMR and DEPT135 spectra of dichotocejpin B (**2**) in DMSO-d_6.

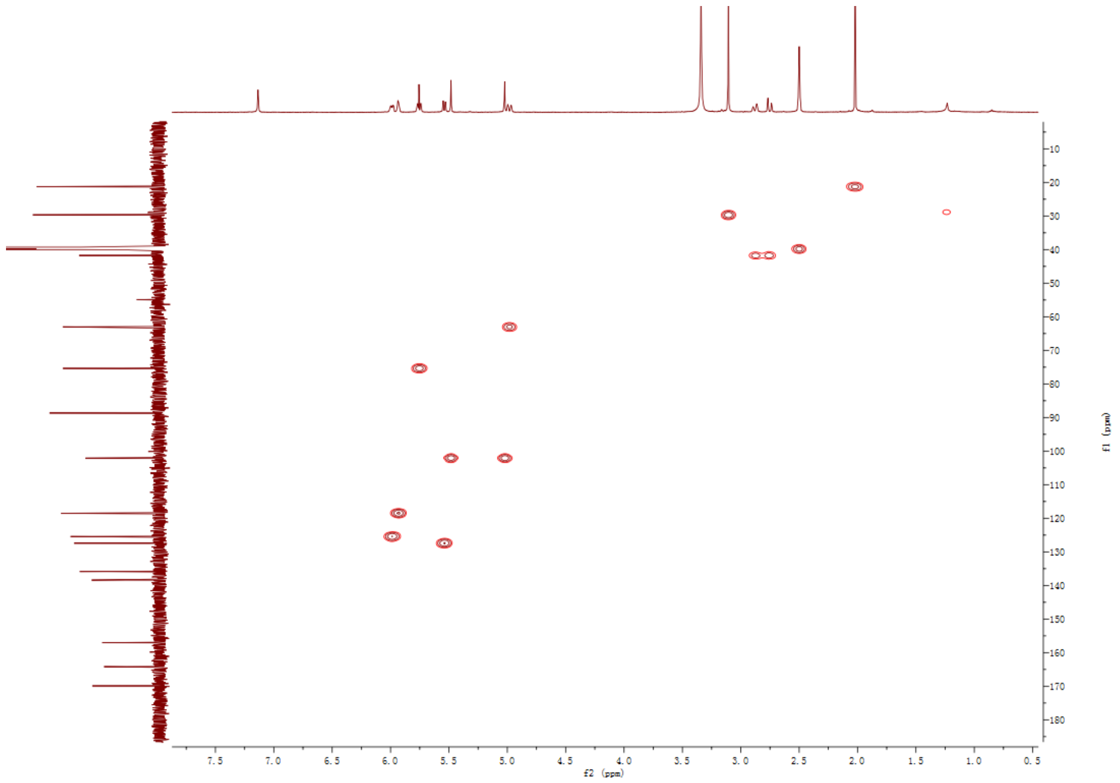

Figure S11. HSQC spectrum of dichotocejpin B (**2**) in DMSO-d_6.

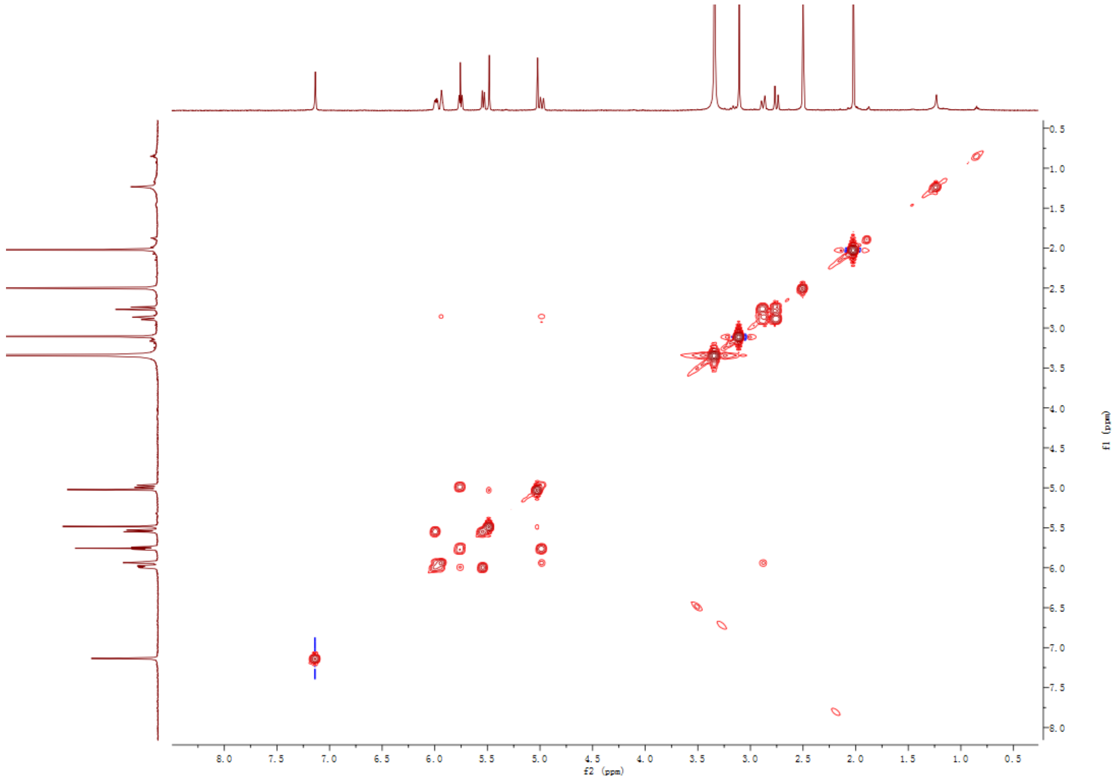

Figure S12. ^1H–^1H COSY spectrum of dichotocejpin B (**2**) in DMSO-d_6.

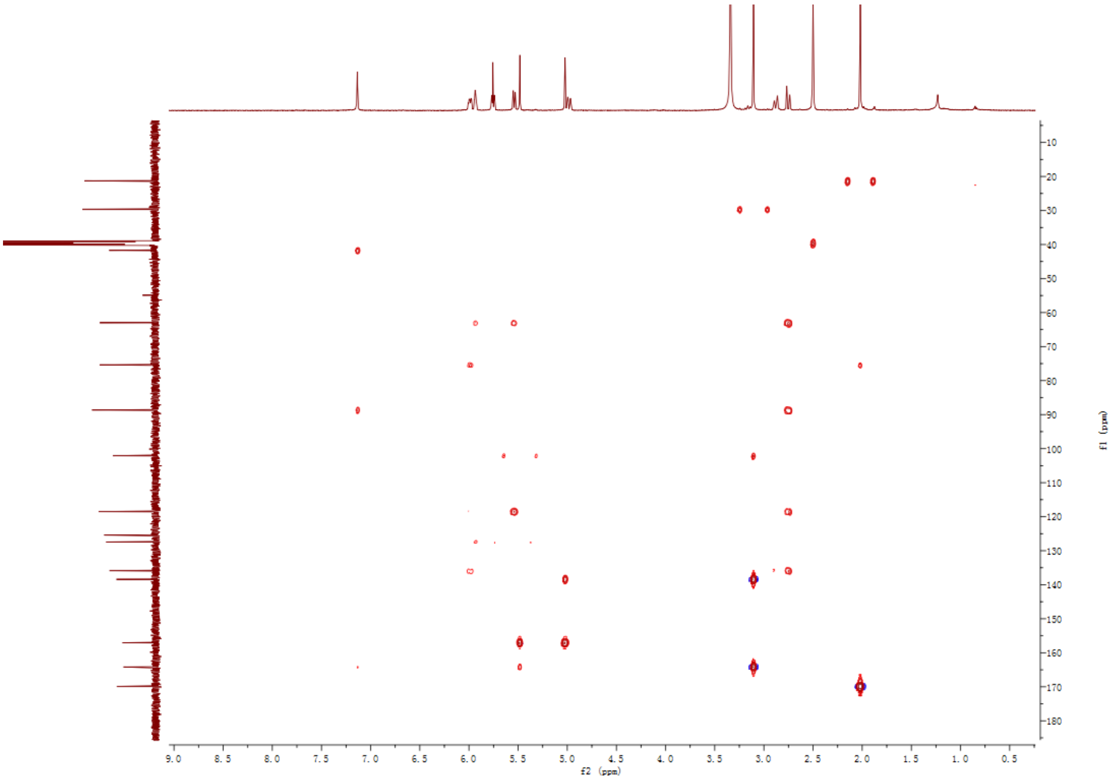

Figure S13. HMBC spectrum of dichotocejpin B (**2**) in DMSO-d_6.

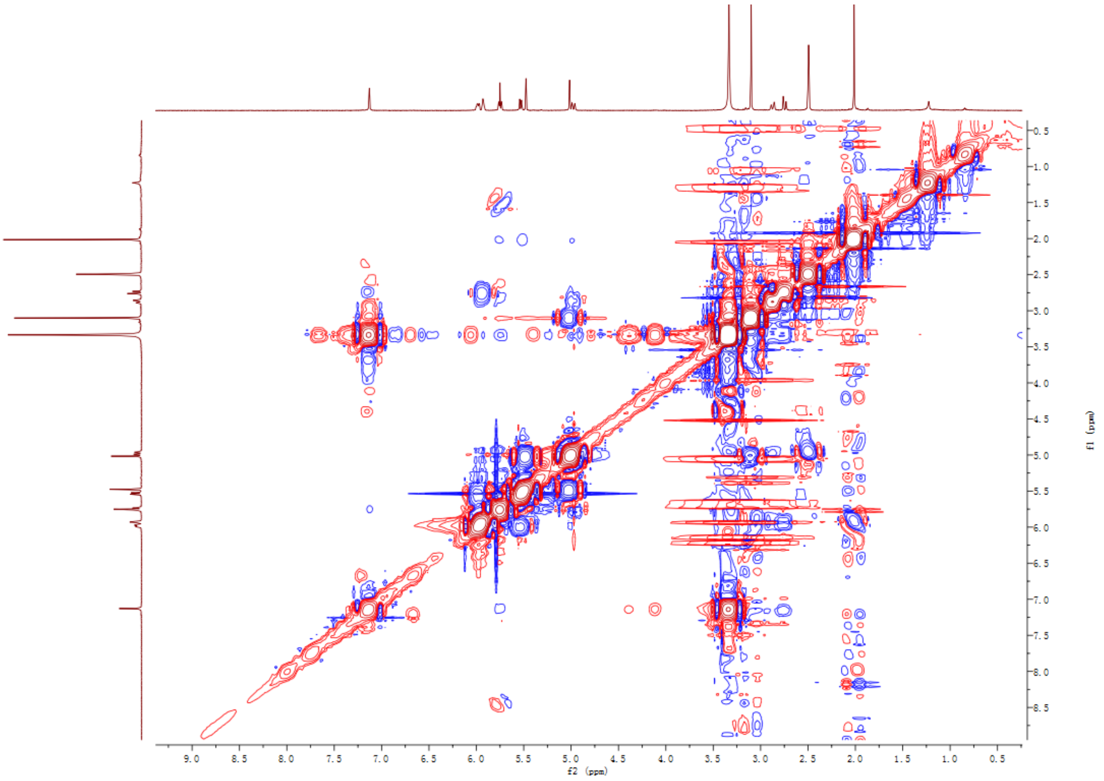

Figure S14. NOESY spectrum of dichotocejpin B (**2**) in DMSO-d_6.

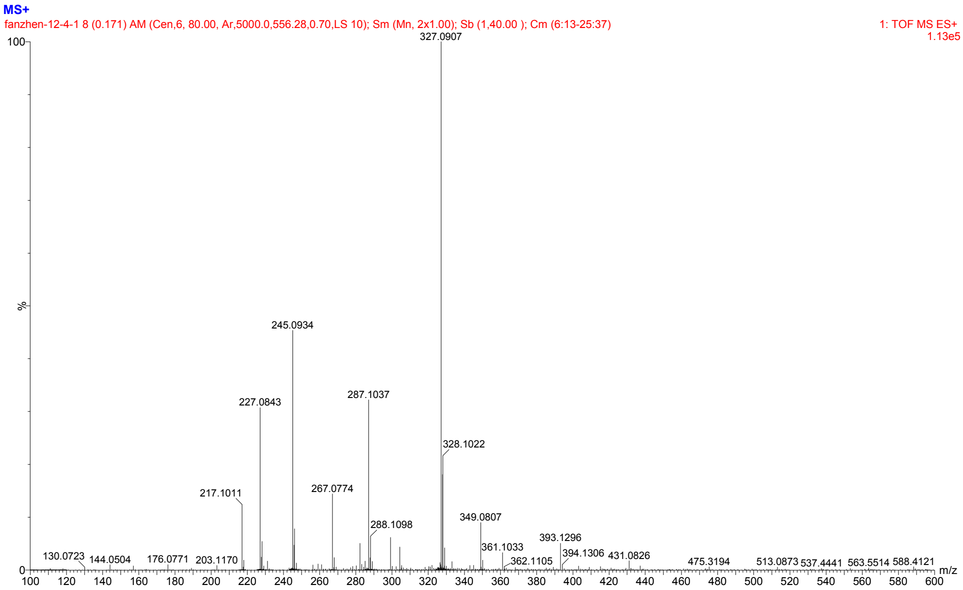

Figure S15. HRESIMS spectrum of dichotocejpin B (**2**).

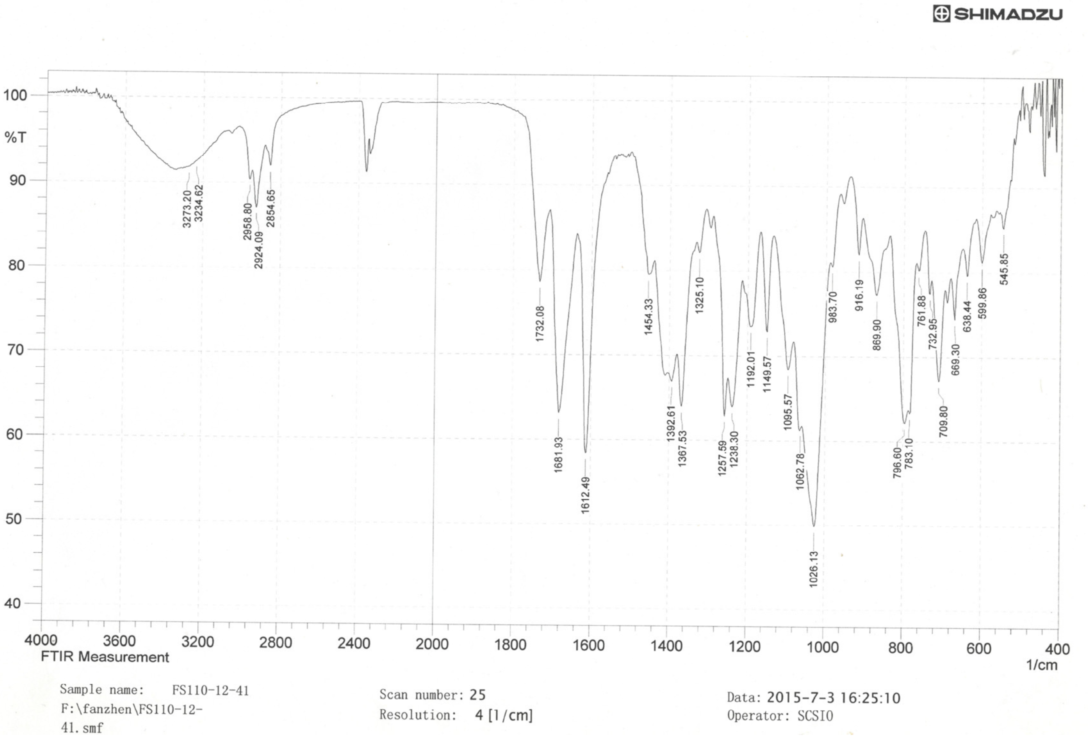

Figure S16. IR spectrum of dichotocejpin A (**2**).

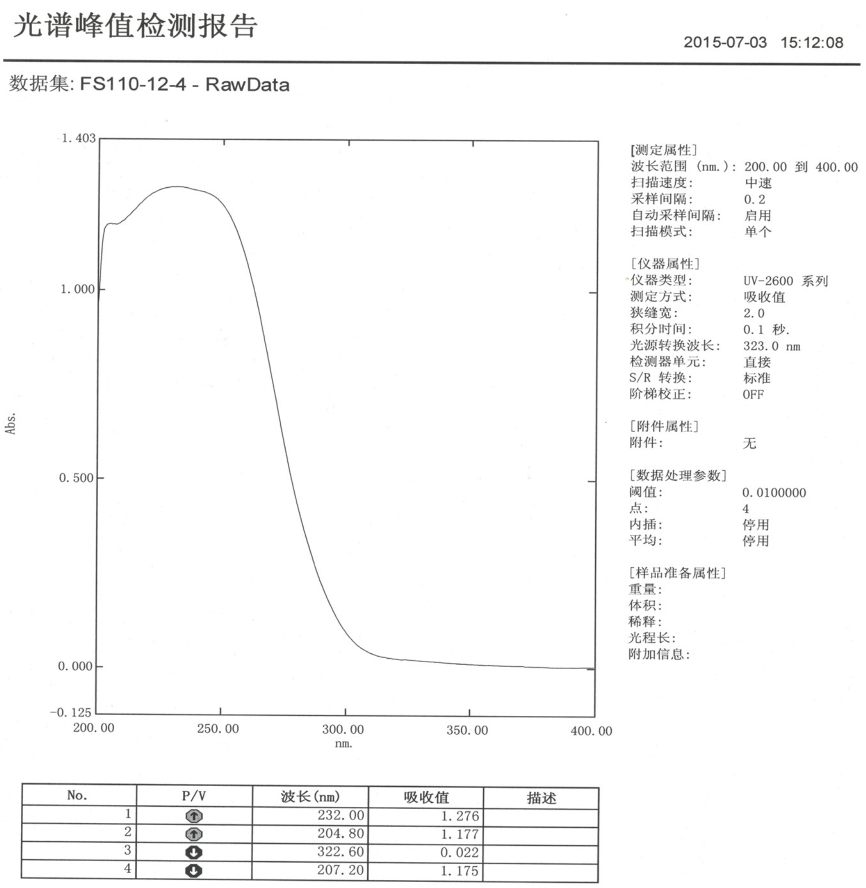

No.	P/V	波长(nm)	吸收值	描述
1	↑	232.00	1.276	
2	↑	204.80	1.177	
3	↓	322.60	0.022	
4	↓	207.20	1.175	

Figure S17. UV spectrum of dichotocejpin A (**2**).

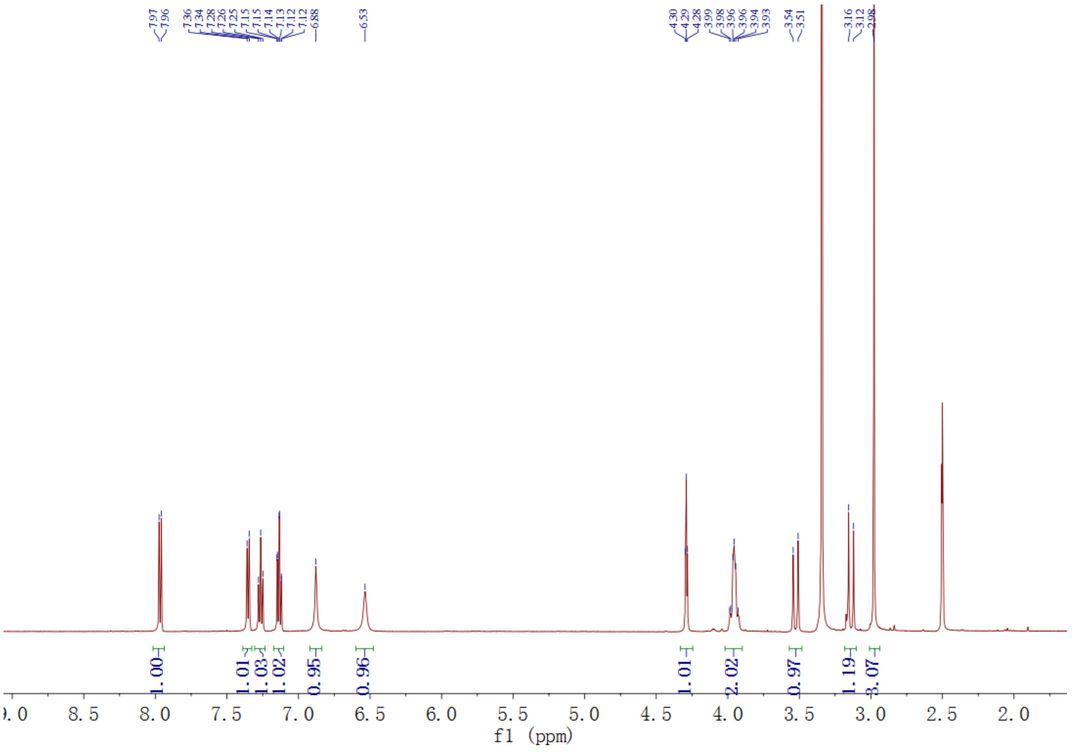

Figure S18. ^{1}H NMR spectrum of dichotocejpin C (**3**) in DMSO-d_6.

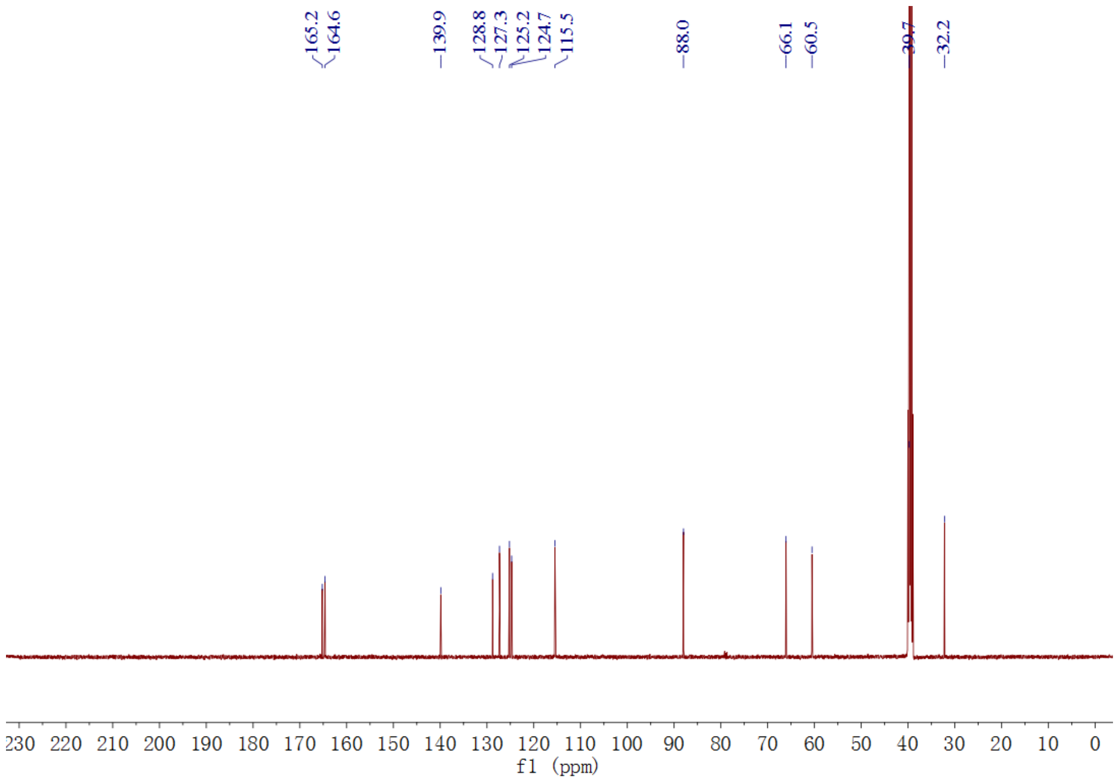

Figure S19. ^{13}C NMR spectra of dichotocejpin C (**3**) in DMSO-d_6.

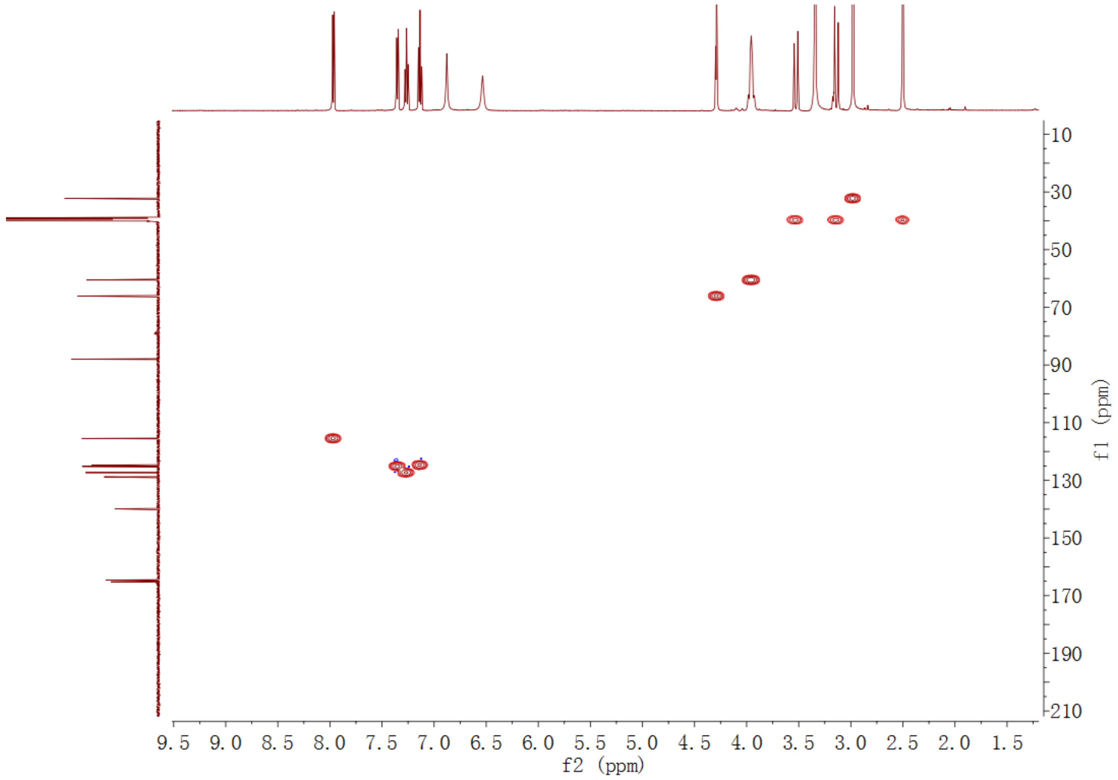

Figure S20. HSQC spectrum of dichotocejpin C (**3**) in DMSO-d_6.

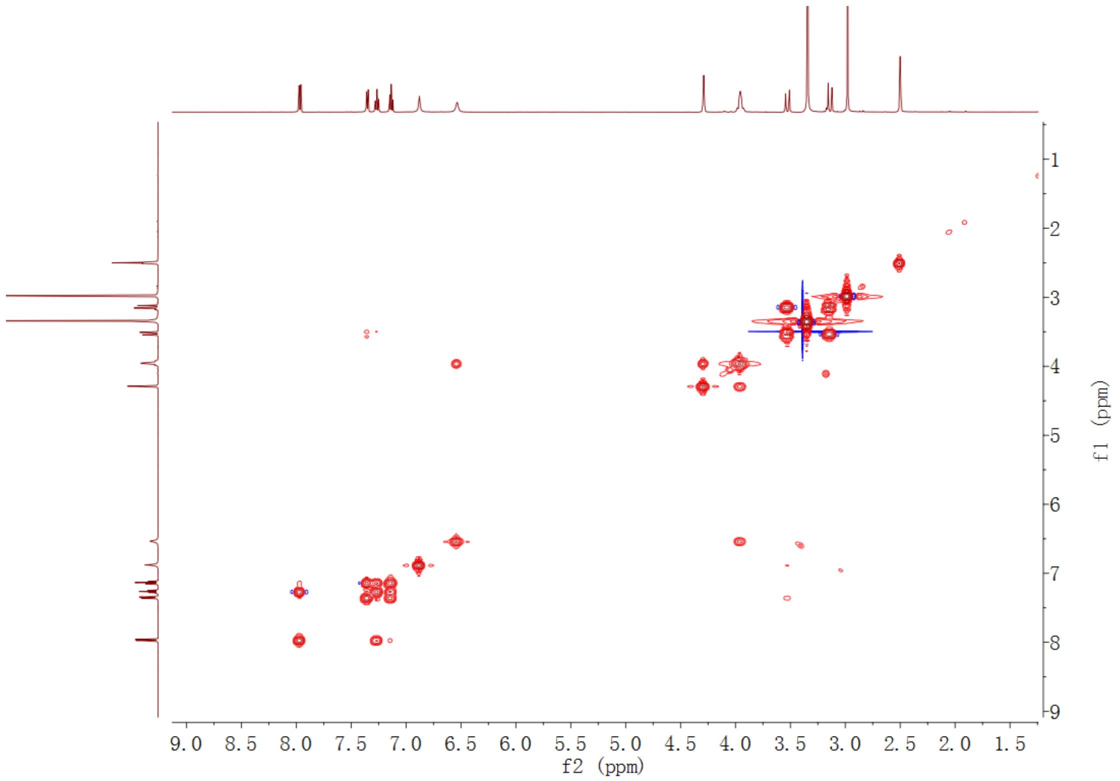

Figure S21. ^1H–^1H COSY spectrum of dichotocejpin C (**3**) in DMSO-*d*$_6$.

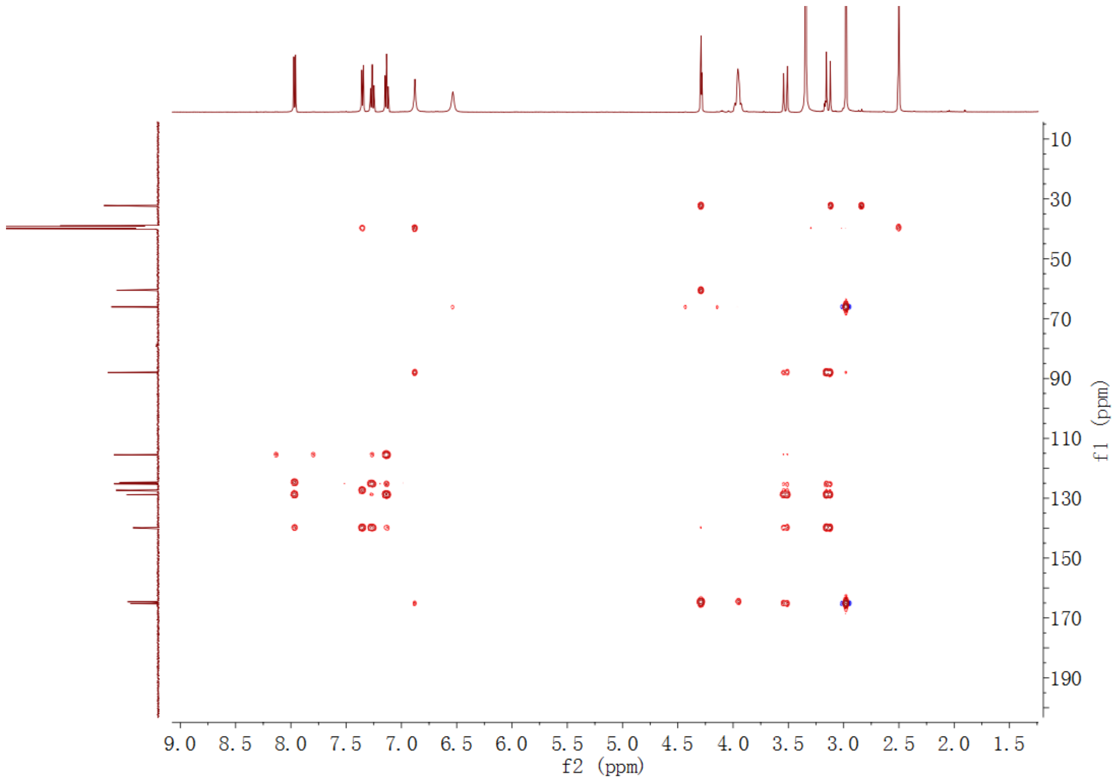

Figure S22. HMBC spectrum of dichotocejpin C (**3**) in DMSO-*d*$_6$.

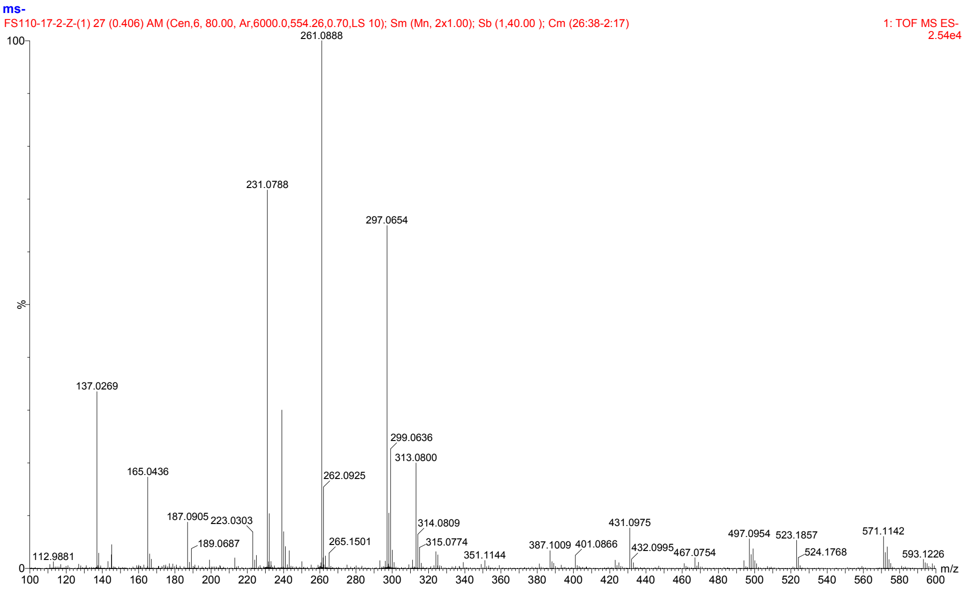

Figure S23. HRESIMS spectrum of dichotocejpin C (**3**).

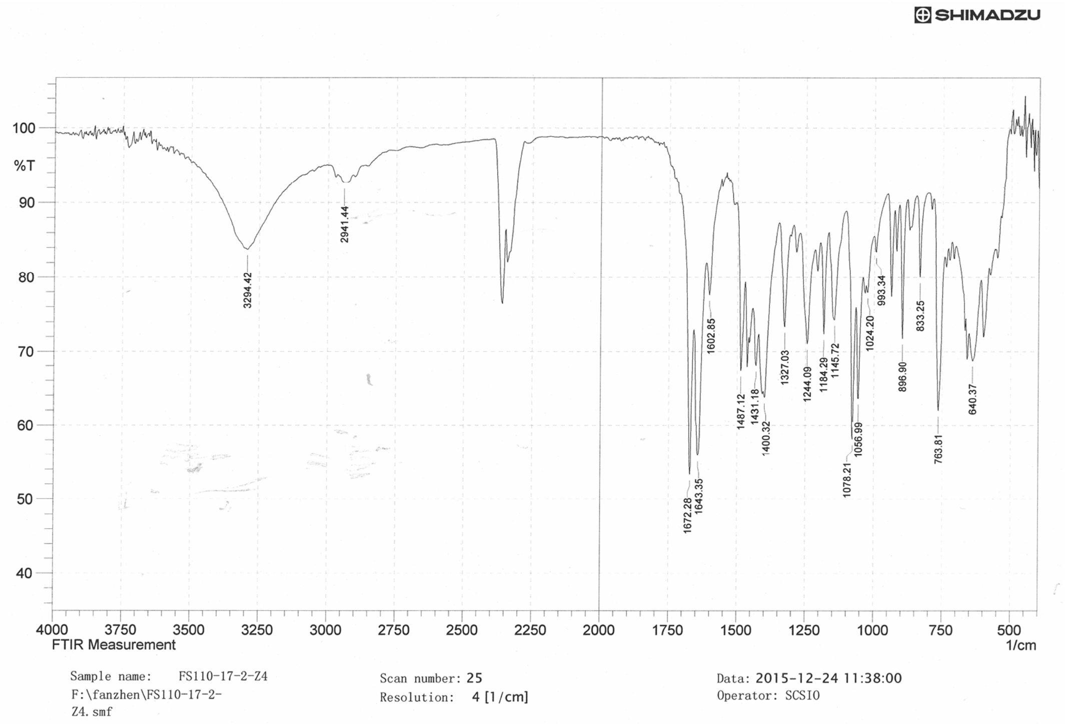

Figure S24. IR spectrum of dichotocejpin A (**3**).

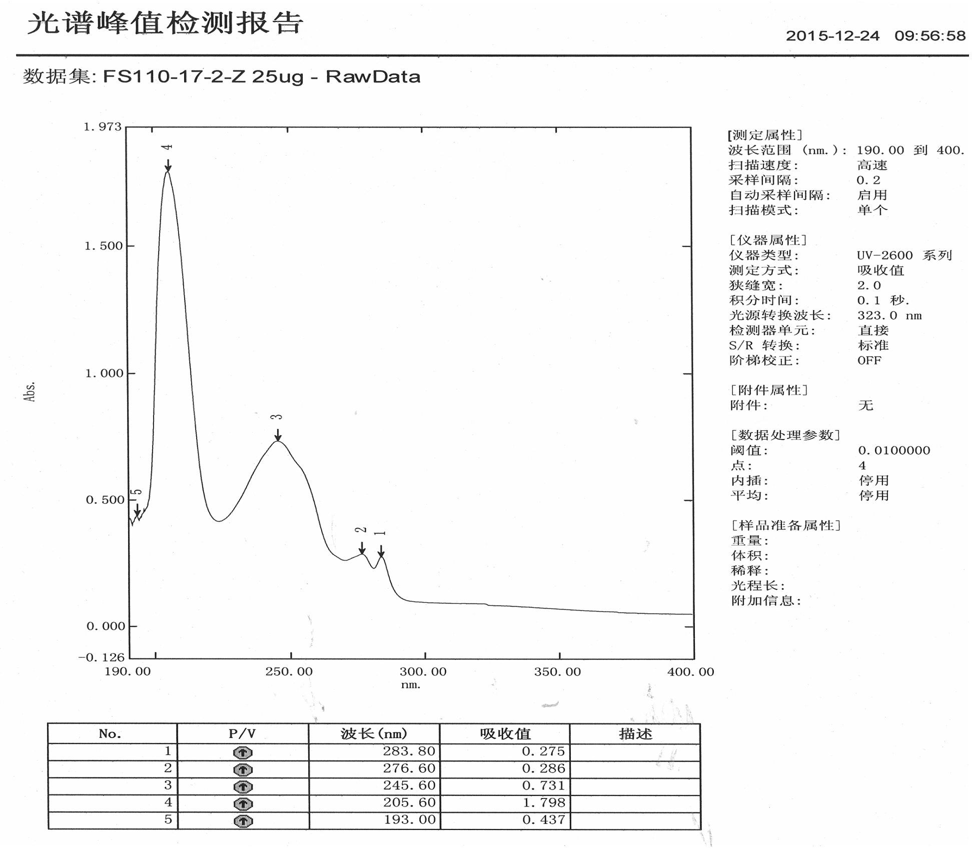

No.	P/V	波长(nm)	吸收值	描述
1	⊕	283.80	0.275	
2	⊕	276.60	0.286	
3	⊕	245.60	0.731	
4	⊕	205.60	1.798	
5	⊕	193.00	0.437	

Figure S25. UV spectrum of dichotocejpin A (**3**).

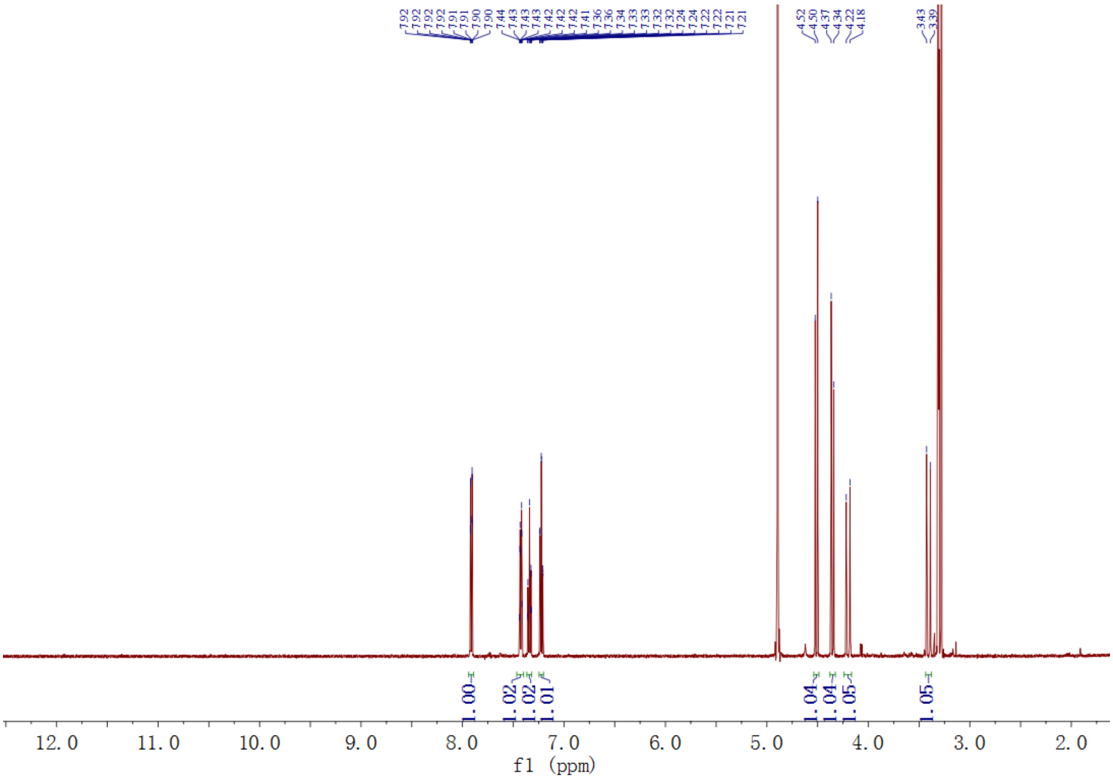

Figure S26. ^{1}H NMR spectrum of compound **4** in CD_3OD.

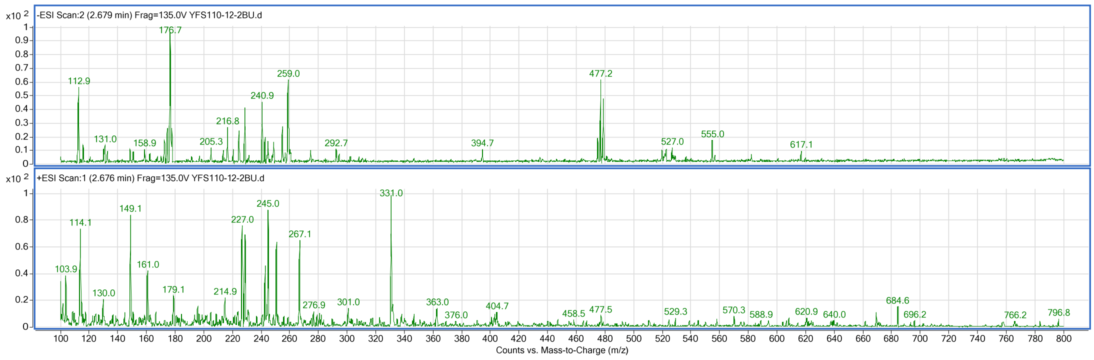

Figure S27. ESI-MS spectrum of compound **4**.

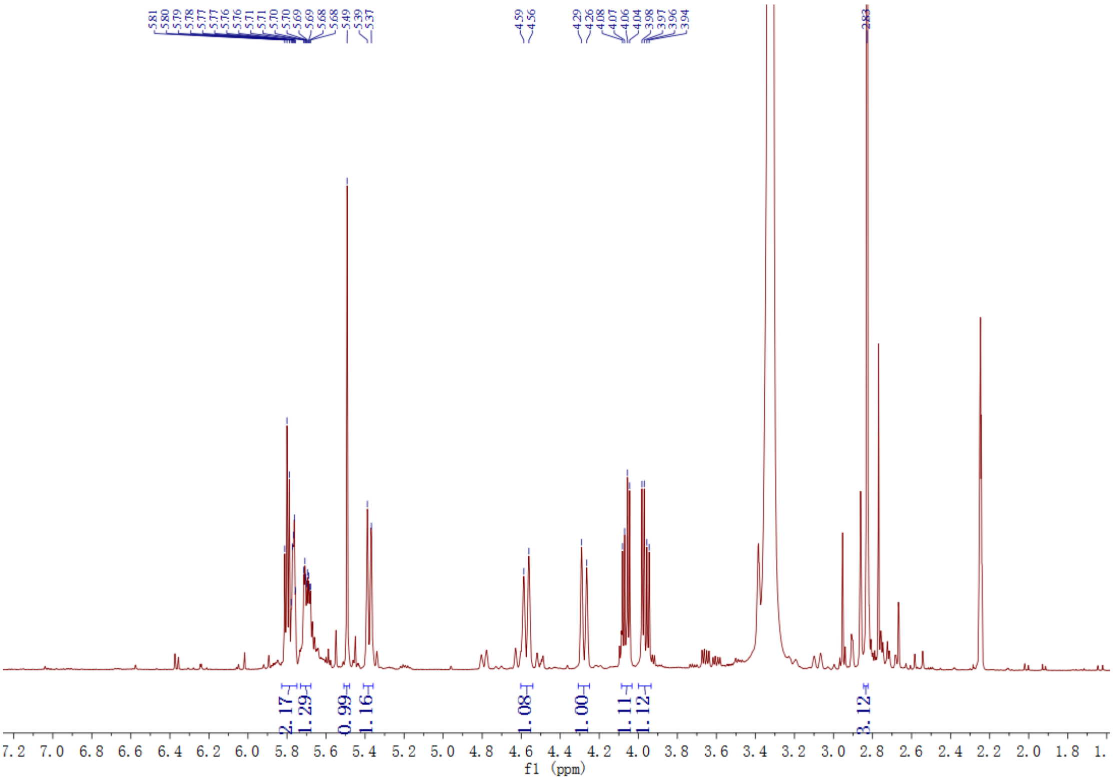

Figure S28. ^{1}H NMR spectrum of compound **5** in CD_3OD.

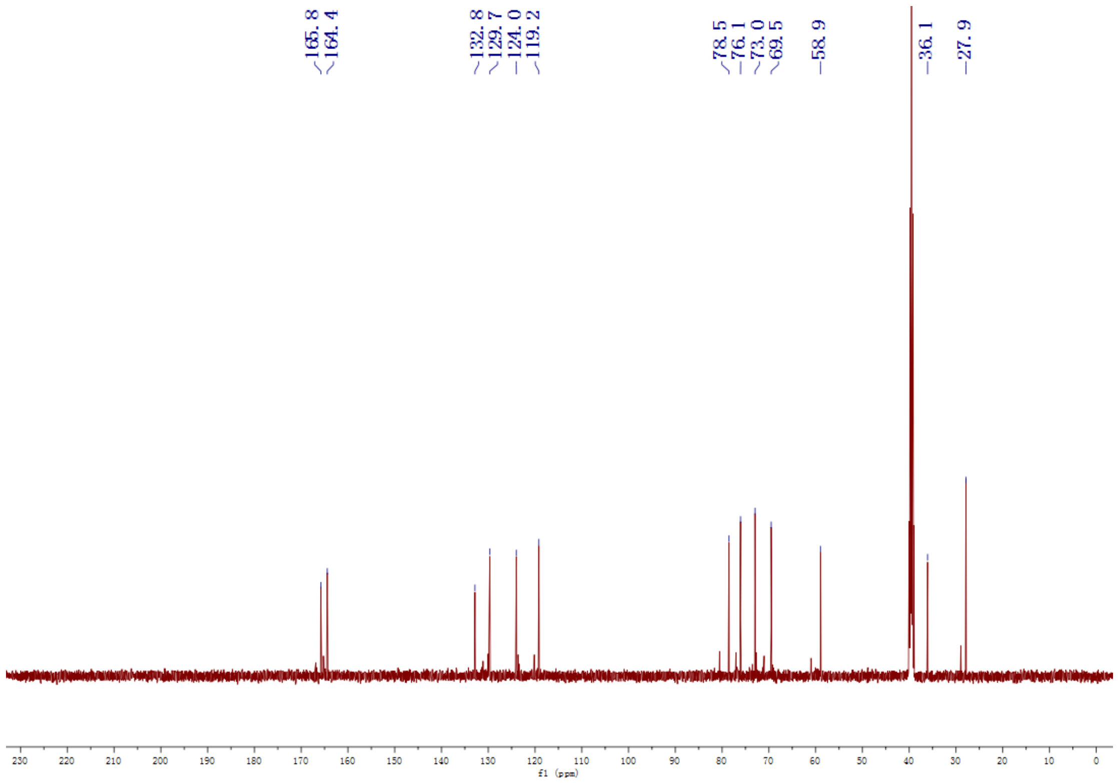

Figure S29. ^{13}C NMR spectrum of compound **5** in CD_3OD.

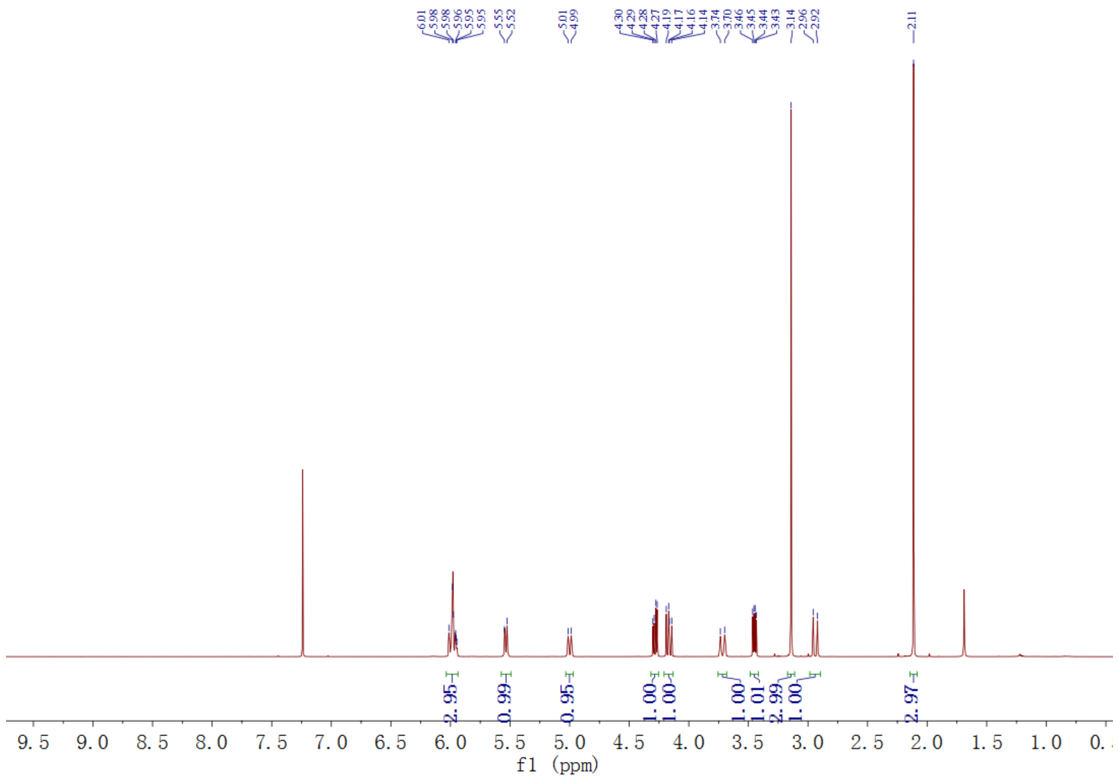

Figure S30. ^{1}H NMR spectrum of compound **6** in $CDCl_3$.

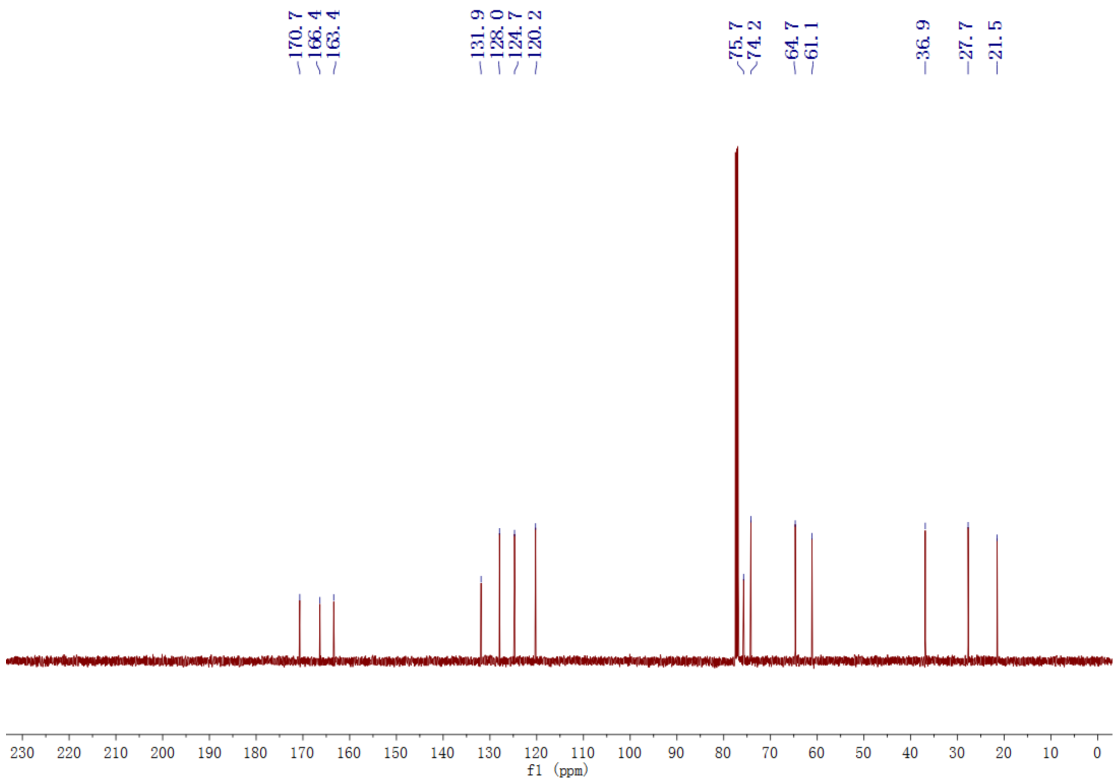

Figure S31. ^{13}C NMR spectrum of compound **6** in $CDCl_3$.

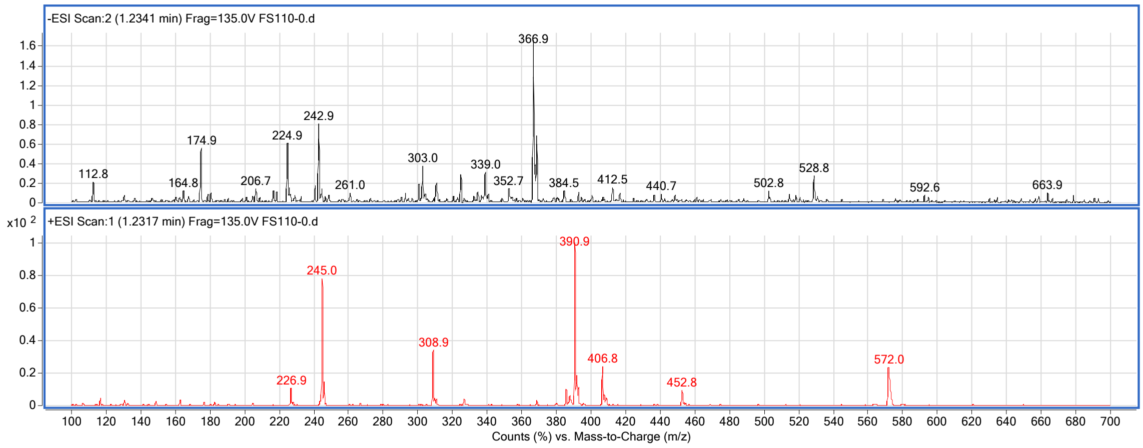

Figure S32. ESI-MS spectrum of compound **6**.

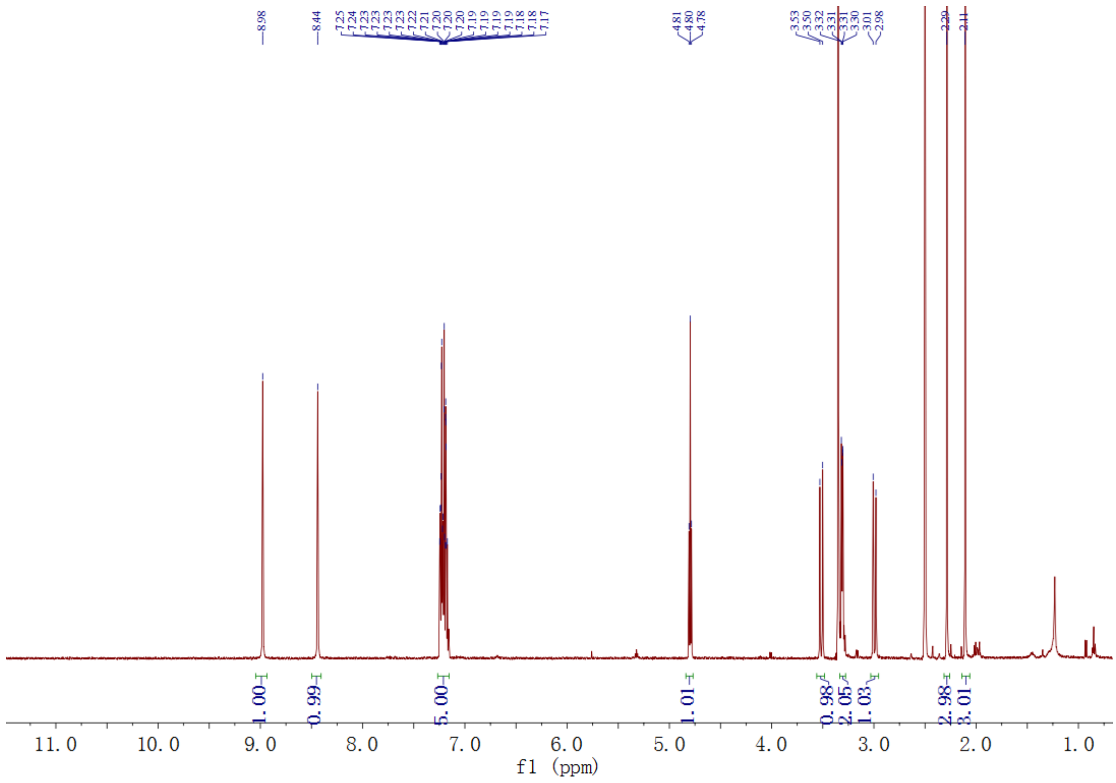

Figure S33. ^{1}H NMR spectrum of compound **7** in DMSO-*d*6.

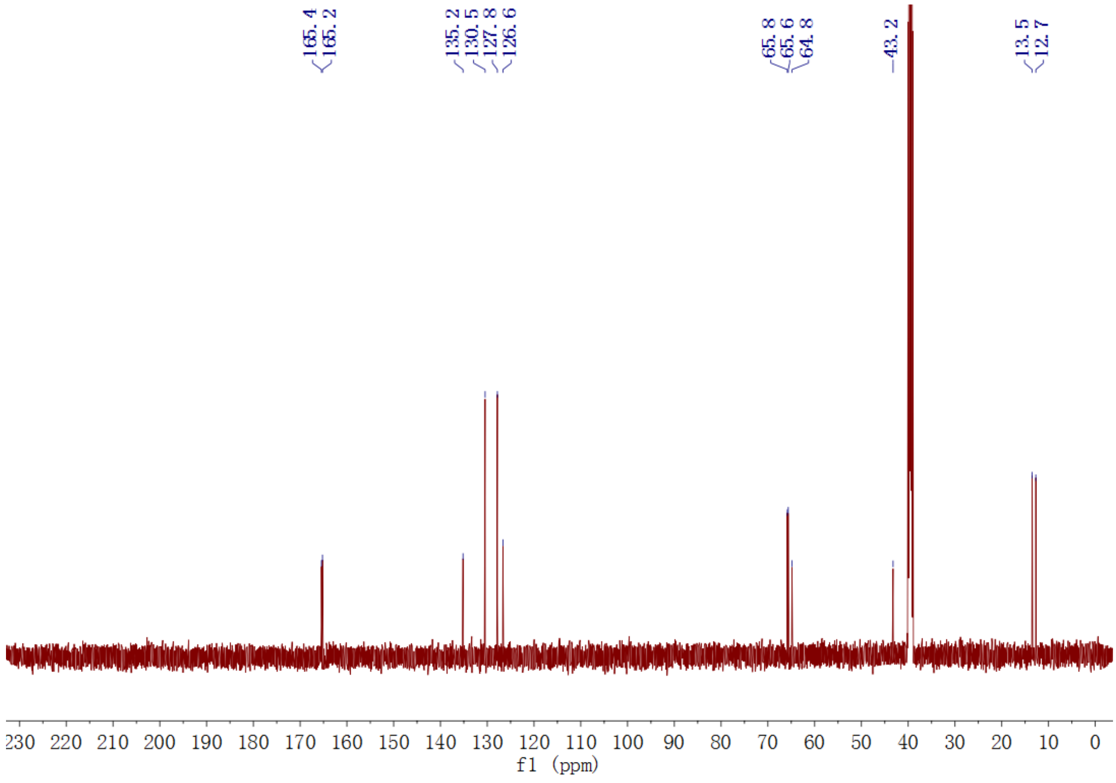

Figure S34. ^{13}C NMR spectrum of compound **7** in DMSO-*d*6.

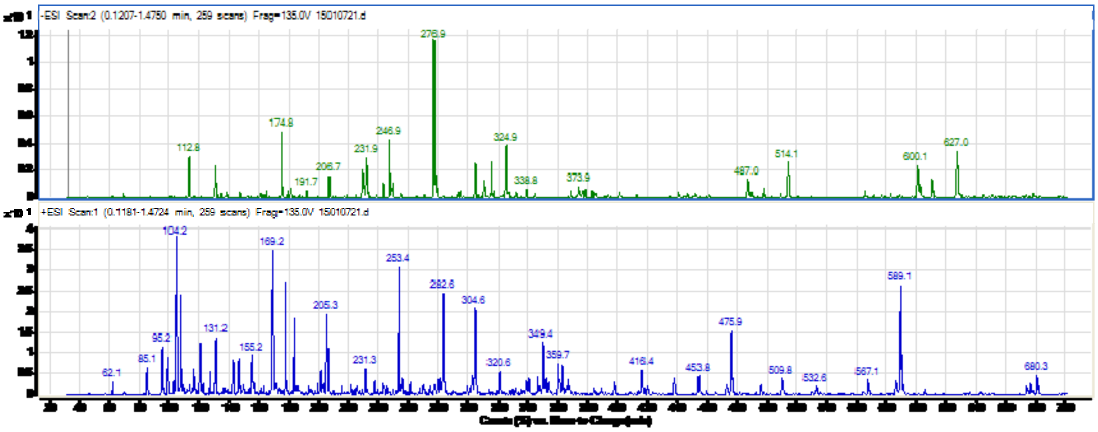

Figure S35. ESI-MS spectrum of compound **7**.

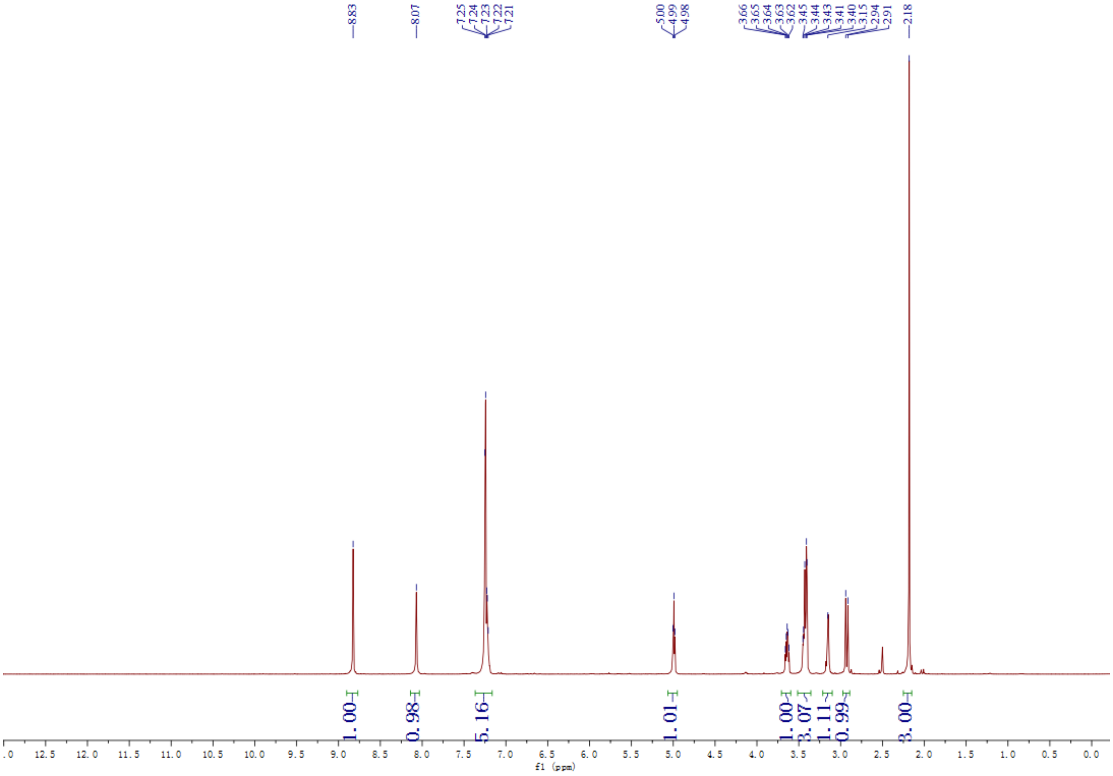

Figure S36. ^{1}H NMR spectrum of compound **8** in DMSO-d_6.

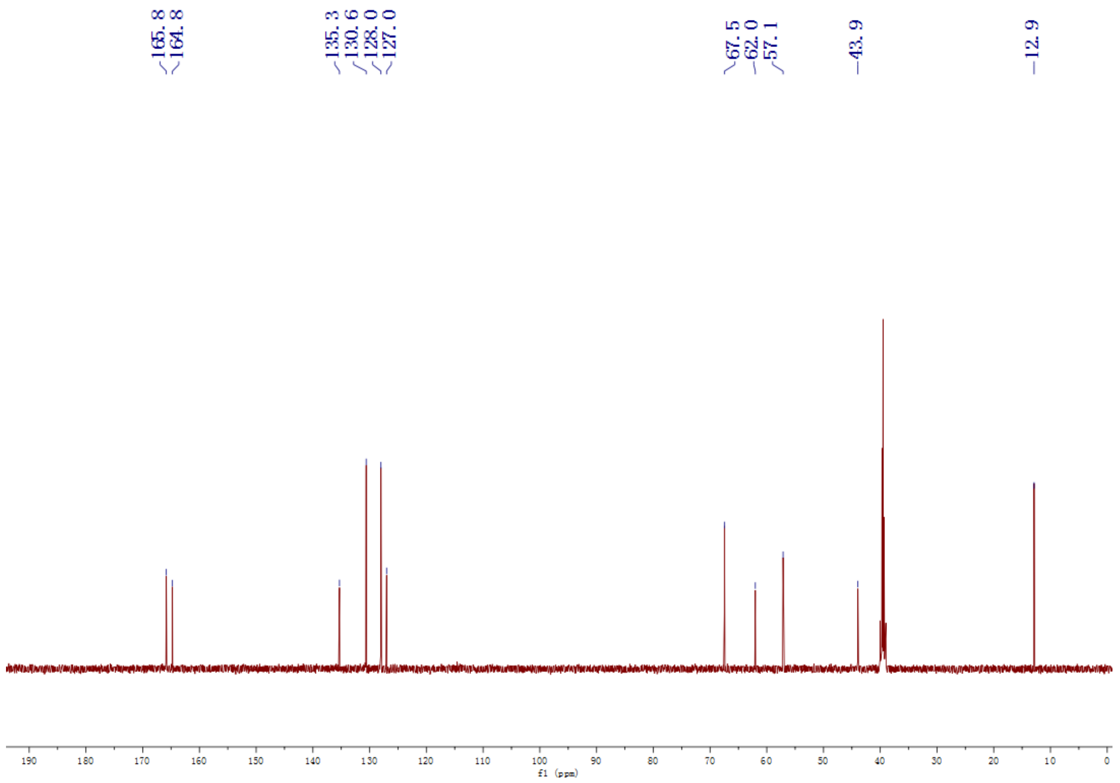

Figure S37. ^{13}C NMR spectrum of compound **8** in DMSO-d_6.

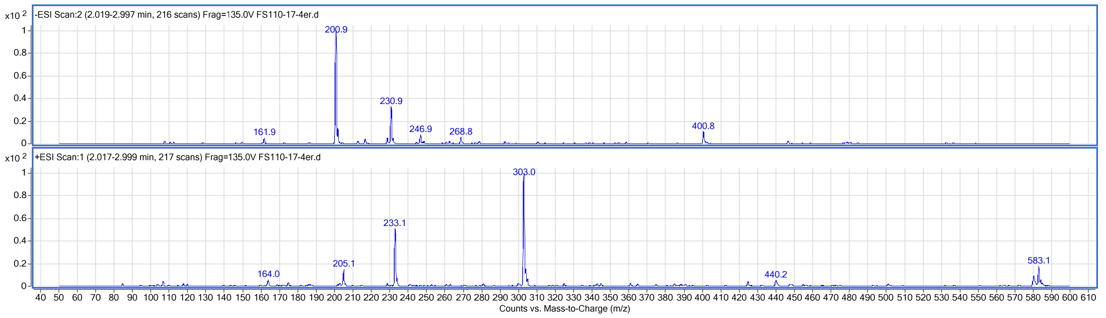

Figure S38. ESI-MS spectrum of compound **8**.

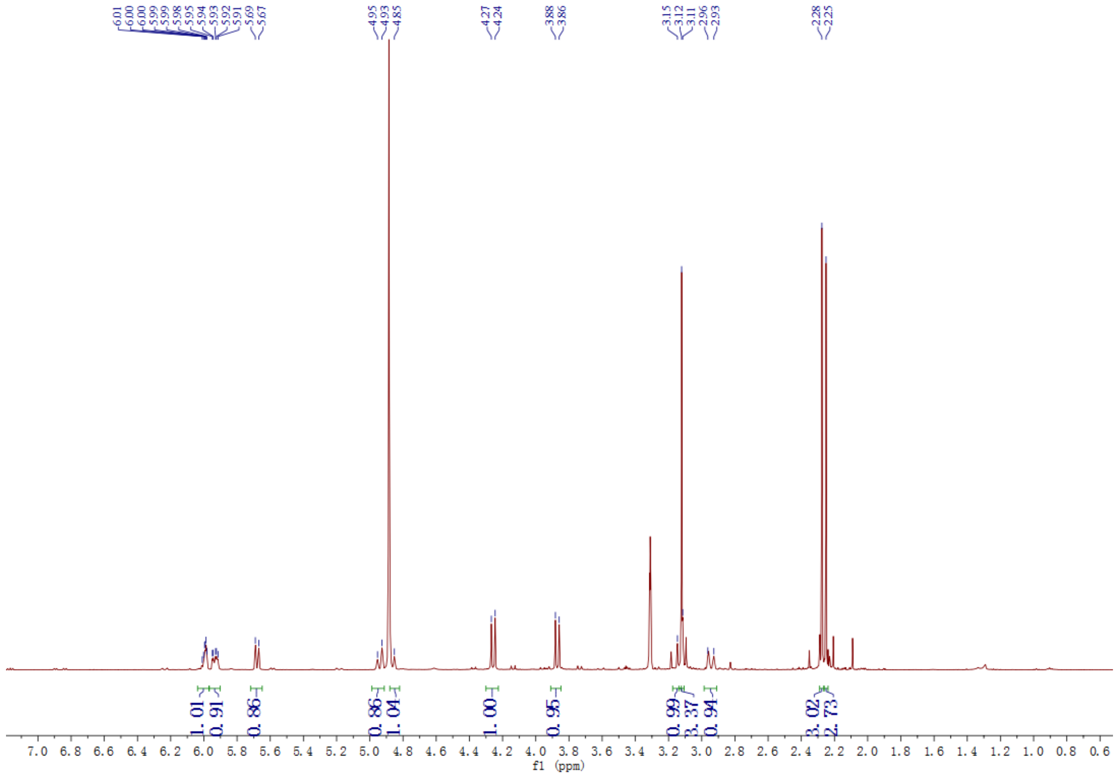

Figure S39. ^{1}H NMR spectrum of compound **9** in CD_3OD.

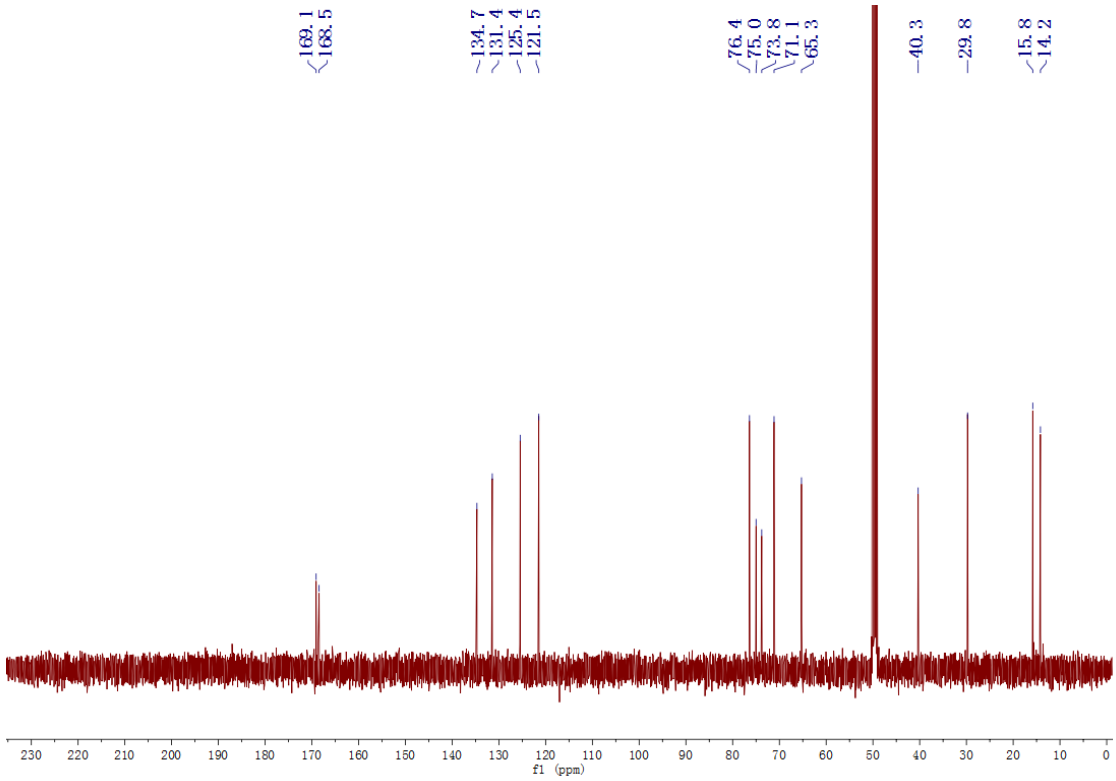

Figure S40. ^{13}C NMR spectrum of compound **9** in CD_3OD.

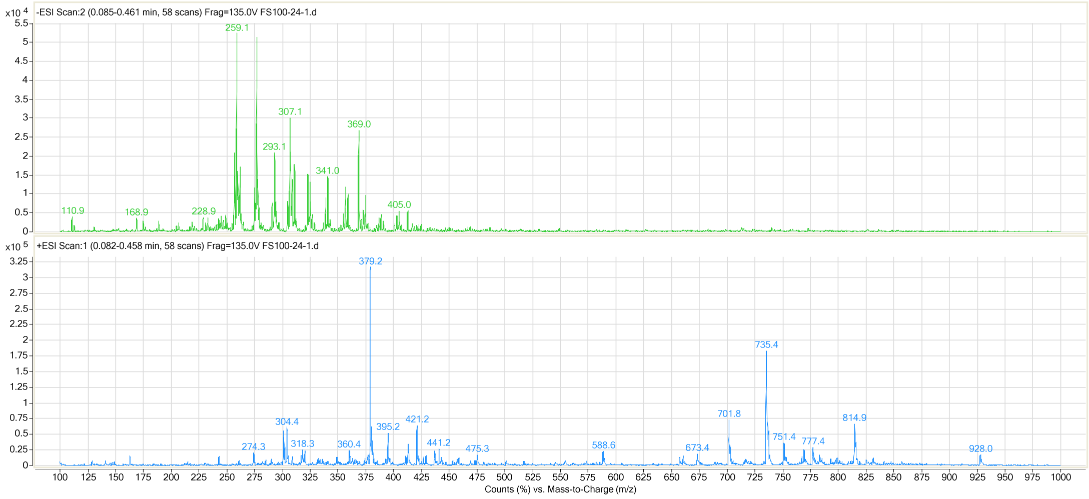

Figure S41. ESI-MS spectrum of compound **9**.

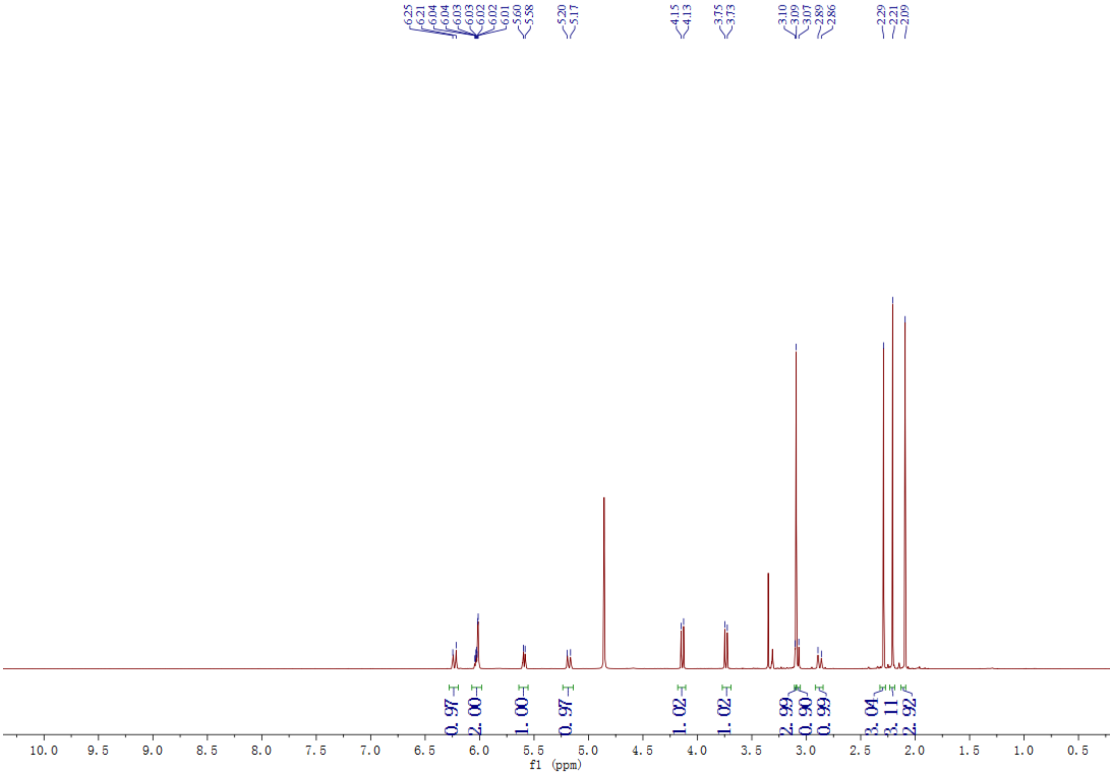

Figure S42. ^{1}H NMR spectrum of compound **10** in CD_3OD.

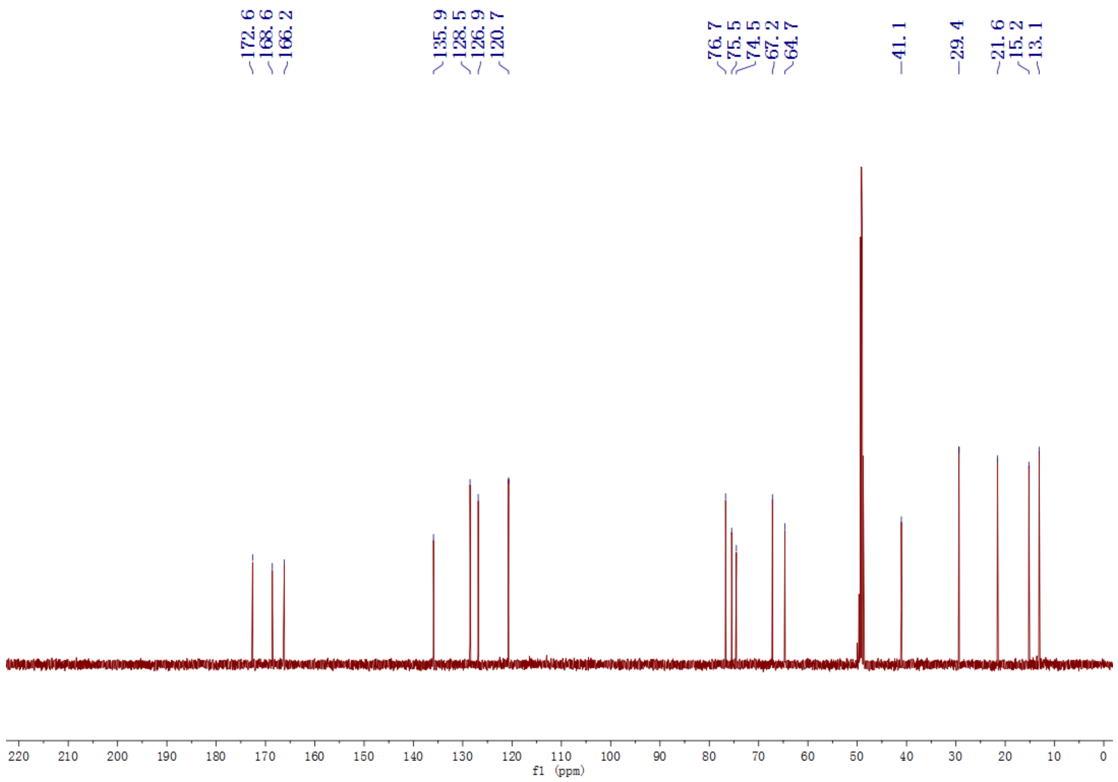

Figure S43. ^{13}C NMR spectrum of compound **10** in CD_3OD.

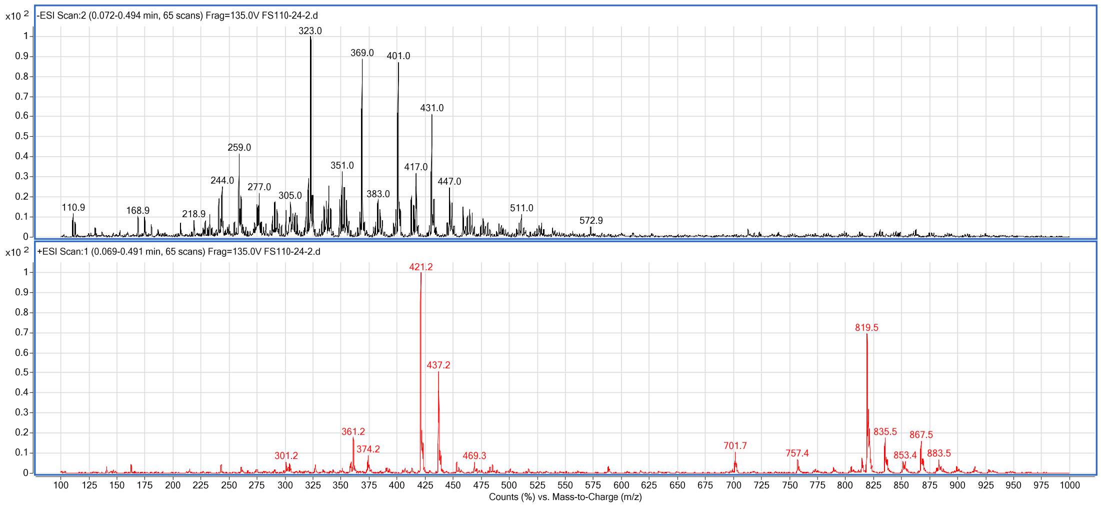

Figure S44. ESI-MS spectrum of compound **10**.

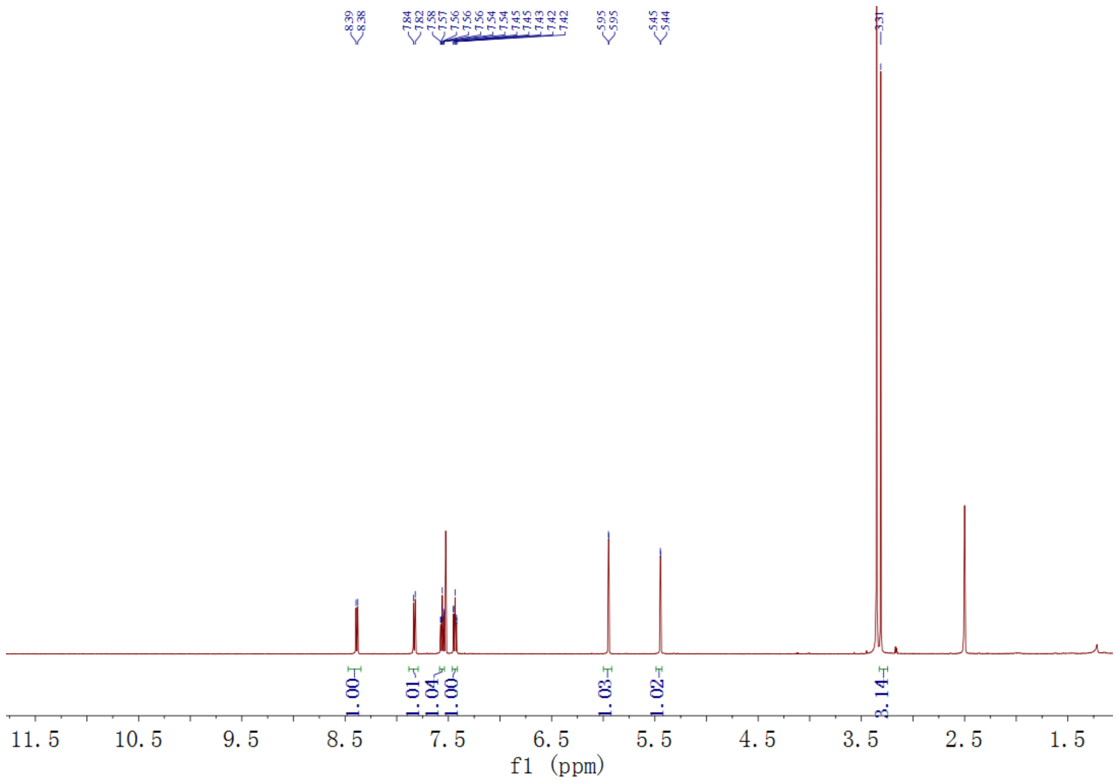

Figure S45. ^{1}H NMR spectrum of compound **11** in DMSO-d_6.

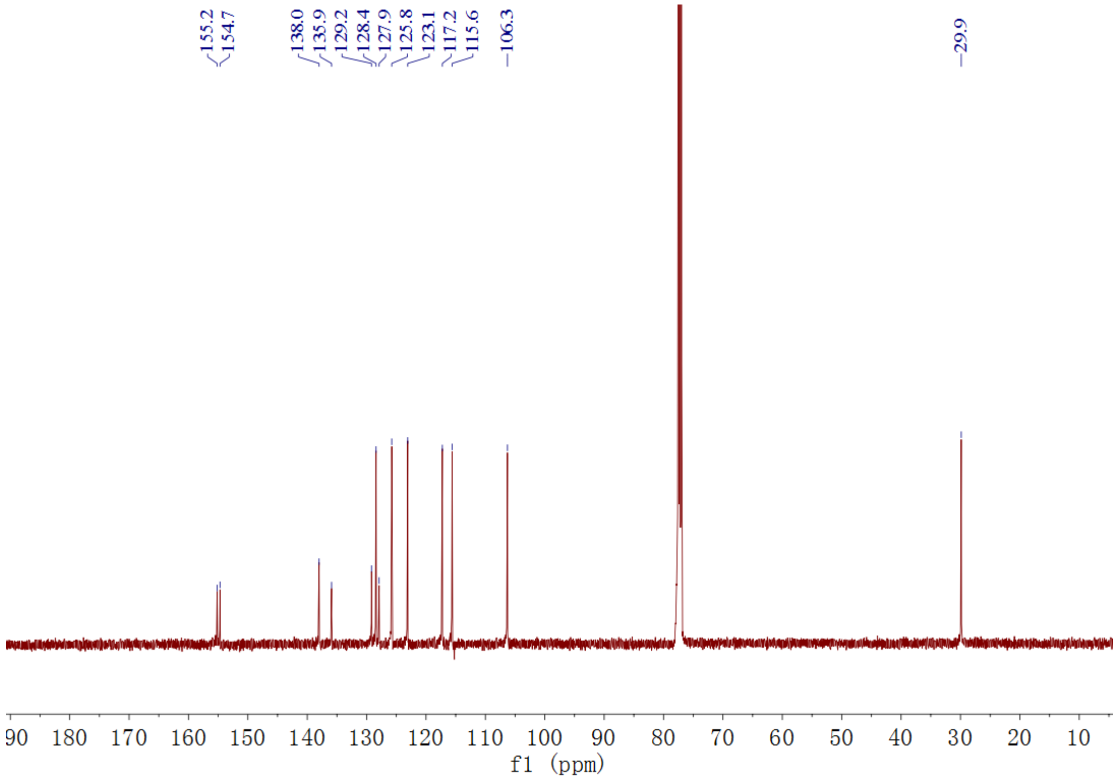

Figure S46. ^{13}C NMR spectrum of compound **11** in $CDCl_3$.

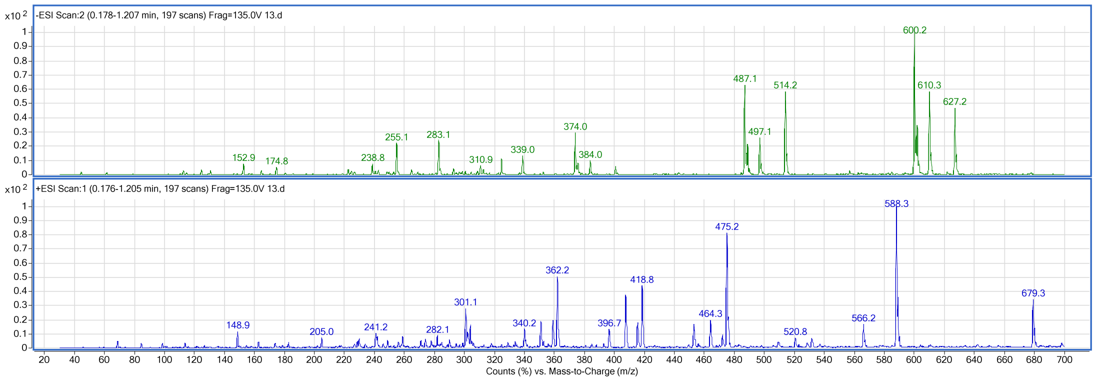

Figure S47. ESI-MS spectrum of compound **11**.
